# Systematic Review and Meta-Analysis of Quality Claims Associated with Fresh Pet Food: Evaluating Scientific Evidence for Additives, Ingredient Quality, and Effects of Processing in Pet Nutrition

**DOI:** 10.3390/ani16010041

**Published:** 2025-12-23

**Authors:** Matthew T. Jobe, Kevin M. Downs

**Affiliations:** School of Agriculture, Middle Tennessee State University, Murfreesboro, TN 37132, USA; matthewjobe15@gmail.com

**Keywords:** canine, feed additives, feline, fresh pet food, pet nutrition

## Abstract

The fresh pet food market has experienced substantial growth, with manufacturers making quality claims that influence consumer purchasing decisions and veterinary recommendations. This systematic review evaluates the scientific evidence supporting three prevalent claims associated with fresh pet food: that additives, preservatives, and fillers are harmful to pet health; that human-grade ingredients provide superior safety and nutrition compared to feed-grade ingredients; and that whole ingredients offer health advantages over processed ingredients. A comprehensive literature search across the SCOPUS, PubMed, and EBSCO databases identified 4888 potential studies. Following systematic screening and quality assessment, 121 studies met inclusion criteria for analysis. Bayesian meta-analyses of additives (*n* = 60 studies) and preservatives (*n* = 39 studies) revealed pooled risk differences of 0.0006 and 0.0003, respectively, with Bayes factors strongly supporting null hypotheses of no adverse effects within regulatory limits. Random-effects meta-analyses of processing effects on ingredient digestibility (*n* = 102 comparisons, SMD = 1.971, *p* = 0.005) and nutrient content (*n* = 137 comparisons, SMD = 1.405, *p* < 0.001) demonstrated significant heterogeneity, with outcomes highly dependent on ingredient type and processing method rather than processing intensity. Human-grade versus feed-grade ingredient comparisons (*n* = 6 studies) showed methodological limitations and high risk of bias, preventing definitive conclusions. Current evidence does not substantiate claims that approved additives and preservatives cause harm when used within AAFCO guidelines. Processing effects vary substantially by ingredient matrix and method, with both beneficial and detrimental outcomes observed. This review identifies critical research gaps and provides recommendations for evidence-based marketing practices, targeted research priorities, and informed decision-making by industry professionals and consumers.

## Simple Summary

Pet owners today face an overwhelming array of dietary options for their companions, from grain-free kibble to fresh-frozen meals and boutique brands, each marketed with claims of superior nutrition and improved health outcomes. Understanding these options and their consequences on pet health is essential for both consumers making purchasing decisions and veterinary professionals recommending dietary formats. Beyond basic nutritional adequacy, consumers must evaluate product-related factors including the presence of additives, preservatives, and fillers; the use of human-grade ingredients; processing temperatures and methods; whole versus processed ingredients; and overall nutrient integrity. This systematic review examined three prevalent quality claims made by leading fresh pet food companies: that additives and preservatives are harmful, that human-grade ingredients offer superior safety and nutrition, and that whole ingredients provide greater health benefits than processed alternatives. We assessed the scientific evidence supporting each claim and evaluated their potential health impacts on dogs and cats.

## 1. Introduction

According to an industry analysis, the global pet food market achieved a value of $126.66 billion in 2024, with the fresh pet food segment representing $1.51 billion of this total market share [1,2]. This emergence into the market is not coincidental and portrays the consumer demand for their pets to consume more recognizable food that resembles a human diet. It is apparent that modern pet owners have a higher standard for pet food quality and aim to hold the industry to a higher standard [3]. Previously, pet owners considered home-cooked and human-grade pet foods to be luxury items because of their high cost and complicated manufacturing process. However, improved production efficiency and the rise of direct-to-consumer sales have fundamentally changed this perception. Therefore, there is an increasing demand for fresh pet food. With studies showing the associations between processed food intake and negative health outcomes, consumers are likely deterred from unrecognizable options, such as kibble or canned pet foods [4,5,6]. The quantity of pet food brands, formats, and claims has created difficulties, with 25% of owners feeling overwhelmed and 50% reporting that selecting the right pet food is the hardest part of pet ownership [7].

Before exploring the impact of fresh pet food (FP) on pet health, it was necessary to define the term ‘fresh’ in the context of pet food. With visible chunks of identifiable ingredients and familiar aromas, FP generally resembles human-grade food in preparation and quality. Fresh pet food is typically prepared using minimal processing techniques such as steaming, roasting, and air-drying to preserve nutrients [8,9,10,11,12,13,14,15,16,17,18]. These methods contrast with commercial methods such as sterilization via retort or thermal processing via extrusion and high-temperature drying. These pet foods are commonly stored in refrigerated or frozen conditions due to a lack of additives or preservatives which are known to provide shelf stability at ambient temperatures. The Centers for Disease Control and Prevention (CDC) describe FP as “refrigerated, cooked with fewer or no preservatives, and shorter shelf life” than kibble or canned pet foods [19]. Fresh pet food has no definition from the Association of American Feed Control Officials (AAFCO), requiring a market search to better understand its differentiating qualities from common processed pet food formats such as kibble, loaf, pâté, and chunks in gravy (Section 2.1).

Multiple steps are required to transform pet food ingredients into safe, complete-and-balanced, and palatable final products. Commercial pet food is manufactured using distinct processing pathways which are optimized for the given format. Dry kibble production begins with grinding and sieving, followed by precision mixing, preconditioning, and a thermomechanical extrusion process [20]. The extrusion process typically operates at 140–180 °C with high pressures which force extrudate through a die plate with rotary cutting [20]. The drying process controls moisture to <10% while post-extrusion coating supplies fats and palatants [20]. Wet pet food production utilizes a canning process similar to human food which consists of ingredient preparation, grinding, emulsification, mixing, and filling 14). This product is sealed and placed into a retort at setpoints typically around 116–129 °C for 20–100 min [21]. These production processes allow manufacturers to inhibit microbial growth, mitigate pathogen risks, and ensure food safety parameters throughout production [22]. From an operational standpoint, this reduces food waste, protects businesses from intentional adulteration, and improves financial performance [23]. Many ingredients in typical pet foods are processed to provide a consistent and safe product before arriving at pet food production sites. This could be in the form of grinding to reach the desired particle size, mixing to achieve homogeneity of nutrients, filtering to remove foreign material, dehydration to control water activity, or washing to remove contaminants. To ensure ingredient safety and quality, pet food companies typically require suppliers to meet specific criteria for parameters including proximate analyses (protein, fat, moisture, and ash), pathogens, mycotoxins, heavy metals, foreign material, and vitamin/mineral concentrations. These parameters are commonly verified through additional testing at production facilities to confirm supplier results and ensure compliance with food safety standards throughout transportation and handling. Different pet food categories employ varying degrees of processing to achieve food safety objectives from extensive thermal processing in shelf-stable products to minimal processing approaches in fresh and frozen formats, each with distinct advantages for pathogen control, shelf stability, and nutrient preservation. The processing approach selected influences product characteristics such as shelf life, distribution requirements, storage conditions, and nutritional profile consistency. Ingredient parameter consistency remains important across all product categories to ensure predictable finished product quality and minimize production variability.

The negative connotation surrounding the processing of ingredients likely results from the impact on human health in recent decades. Lane et al. (2024) performed 45 pooled analyses and found that a greater exposure to ultra-processed foods was associated with a higher risk of negative health outcomes such as type 2 diabetes, cancer, cardiovascular disease, and obesity [4]. While most of this is linked to low-nutrient-density, high-fat, and sugary foods, this has likely deterred consumers from processed pet food (PP) to protect their pets, and thus the demand for less processed formats such as FP has increased. There are some elements to processed pet food that consumers may be unaware of, such as dispersion of essential nutrients, increased bioavailability of fibers, and improved digestibility of certain protein sources due to thermal and mechanical energy [24,25,26]. These elements contribute to the “healthiness” of pet food and thus directly affect the pet’s susceptibility to chronic diseases [27,28,29]. The humanization of pets in recent years has also impacted consumers’ choices to feed their pets more human-grade foods, which may present some risks. Firstly, dogs age approximately six to seven times faster than humans, meaning that nutrition-related health effects and disease development occur on a compressed timeline relative to their lifespan [30]. This accelerated timeline means that nutritional deficiencies or imbalances manifest more rapidly in companion animals, emphasizing the importance of complete and balanced formulations at each life stage [31]. Secondly, human diets are reflective of stomach pH, length of the GI tract, and enzyme compositions, which dictate what can be utilized as a substrate for energy or biochemical processes within the body. Canines and felines contain a more variable gastric pH for bone breakdown and a shorter GI tract than humans [32,33]. Enzymatic profiles also differ between species, with dogs and cats lacking active salivary amylase for oral carbohydrate initiation, though dogs possess pancreatic amylase that enables dietary starch utilization [34]. Cats, as obligatory carnivores, have limited pancreatic amylase activity and reduced hepatic glucokinase expression, resulting in lower tolerance for high-carbohydrate diets compared to facultative-omnivores such as the domesticated dog (Canis familiaris) [35]. Dietary decisions for companion animals should prioritize species-appropriate nutrient profiles and bioavailability rather than anthropomorphic preferences based on ingredient format, processing methods, or visual appeal. Humanization of a pet’s diet should be performed in consultation with veterinary professionals to ensure that a pet’s physiological needs are met. However, this should not diminish the expectation that pet food manufacturers will provide fresh, nutritionally sound products. The purpose of this study was to evaluate the most prevalent claims surrounding fresh pet food and to understand their impact on dog and cat health. This was achieved by performing a systematic review of the top three claims to assess whether current scientific evidence provides enough justification to substantiate these claims. Finally, recommendations will be provided to companies and industry professionals that will pinpoint areas for further investigation. This study evaluated whether the current scientific evidence substantiates claims made by fresh pet food manufacturers.

## 2. Materials and Methods

### 2.1. Market Search: Most Prevalent Fresh Pet Food Claims

**Figure 1:**
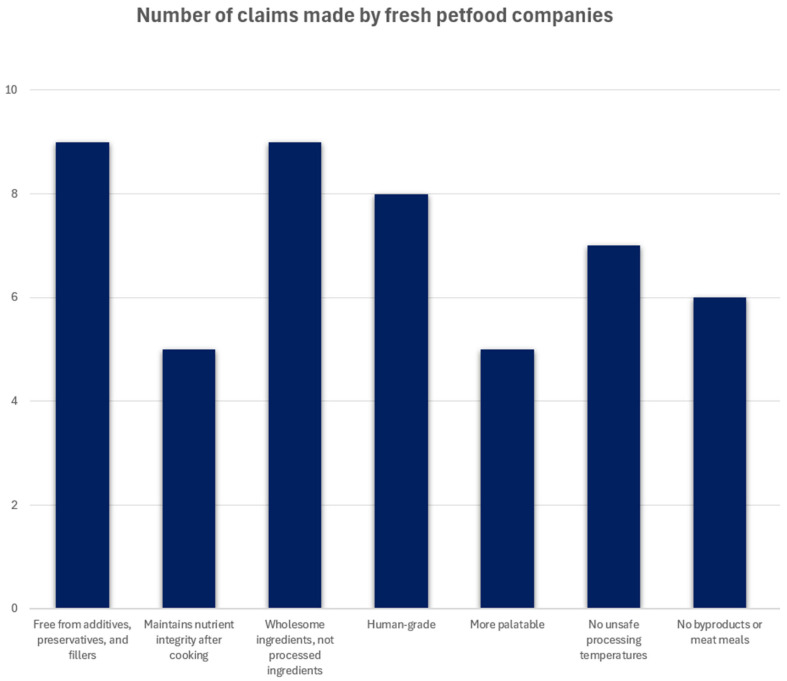
Number of claims made by fresh pet food companies. Note. Market search results across 11 fresh pet food companies [8,9,10,11,12,13,14,15,16,17,18] were recorded and visualized in bar chart format. X-axis reflects specific claims, Y-axis reflects pooled number of claims.

Brands with the highest market share and most reviews were included [36,37,38]. Each brand’s website was searched for claims, which were compiled into a visual representation (see Figure 1). The most prominent claims include the following: “Free from additives, preservatives, and fillers,” “Maintains nutrient integrity after cooking,” “Wholesome ingredients, not processed ingredients,” “Human-grade,” “More palatable,” “No unsafe processing temperatures,” and “No byproducts or meat meals” [8,9,10,11,12,13,14,15,16,17,18]. Amongst those claims, the defining characteristics of FP were the lack of additives, preservatives, fillers; maintaining nutrient integrity after cooking; and the utilization of whole or natural ingredients (see Figure 1). These claims align with pet owners’ desire to provide high-quality and humanized diets for their pets [8,9,10,11,12,13,14,15,16,17,18].

### 2.2. Population, Intervention, Comparison, and Outcome (PICO)

The PICO approach is used in Cochrane reviews to define the scope of a review question through a detailed and consistent strategy [39]. Below are the PICO statements for each of the performed systematic reviews.

#### 2.2.1. Systematic Review of Additives and Preservatives in Pet Food

- Population (P): Dogs and cats of all ages, not limited by health status or geographic location.
- Intervention (I): Exposure to various additives and preservatives in pet food, including the following:
  1. Feed additives (e.g., microalgae species, probiotics, herbal compounds, and medium chain triglycerides);
  2. Preservatives (e.g., antioxidants, antimicrobials, and natural and synthetic preservatives).
- Comparison (C): Control groups receiving diets without the specific additive or preservative, or baseline measurements before intervention.
- Outcome (O): Safety outcomes including adverse reactions, symptoms of toxicity, negative phenotypes, acute toxicity, chronic toxicity, gastrointestinal effects (diarrhea, vomiting), aggression, lethargy, weakness, and death.

#### 2.2.2. Systematic Review of Human-Grade vs. Feed-Grade Ingredients in Pet Food

- Population (P): Dogs and cats of all ages, not limited by health status or geographic location.
- Intervention (I): Complete diets formulated entirely or partially with human-grade ingredients that meet FDA and USDA standards for human consumption, including ingredients approved for human food chain, processed in human food facilities.
- Comparison (C): Complete diets formulated with feed-grade ingredients regulated by the AAFCO and FDA for animal consumption, characterized by different standards for contaminant limits.
- Outcome (O):
  1. Primary: Nutrient levels, contaminant levels (mycotoxins, heavy metals, and pathogens), growth, and metabolic parameters.
  2. Secondary: Digestibility/bioavailability.
  3. Tertiary: Adverse reactions and performance.

#### 2.2.3. Systematic Review of Whole vs. Processed Ingredients in Pet Food

- Population (P): Grains, meat, vegetables, oils/fats, etc.
- Intervention (I): Diets containing whole or minimally processed ingredients in their natural form.
- Comparison (C): Diets or ingredients that have undergone various processing methods including the following:
  1. Thermal processing (extrusion, cooking, roasting, and steaming);
  2. Mechanical processing (milling, grinding, washing, and soaking);
  3. Chemical processing (pH adjustments and preservation methods).
- Outcome (O):
  1. Primary: Nutrient levels, contaminant levels (mycotoxins, heavy metals, and pathogens), growth, and metabolic parameters.
  2. Secondary: Digestibility/bioavailability.
  3. Tertiary: Adverse reactions, performance, antinutrients, bioactive compounds, etc.

### 2.3. Search Strategy

To properly assess the field of research surrounding each claim, a literature search was conducted on research up to February 2025 across three databases: SCOPUS, PubMed, and EBSCO (MEDLINE/Academic Search ultimate). These databases provide extensive coverage of peer-reviewed studies covering a wide array of fields, including veterinary science, animal health, science, and nutrition. No date restrictions were applied to the search so that both early and recent developments in pet food formulations and pet health could be captured, allowing for a comprehensive understanding of the field. To obtain the most applicable studies, the term related to the claim was searched and the following criteria were applied to the search: all open access articles/full-text, English language only, keywords “canine, feline, dog, cat,” article types limited to “data paper, review, and article.” The following criteria from the Cochrane Handbook for Systematic Reviews of Interventions were utilized to conduct this study: PICO domains, predefined and unambiguous eligibility criteria, justifying choice of study design, inclusion of randomized studies, tabulation of extracted data, synthesis of studies, and unbiased reporting [39].

### 2.4. Selection of Studies

The title of the study was analyzed for mentions of key terms related to the claims such as “Additive,” “Preservative,” “Filler,” “Whole Ingredient,” “Processed Ingredient,” “Human-grade,” “Feed-grade,” or any mention of a compound and the effect on a food safety outcome (e.g., microalgal species in fecal metabolites). The utilization of truncation enabled each search query to include variations of a given word. Each systematic review was limited to sources that contained an available full text. The source type was also limited to academic journals, reviews, and reports to retrieve the most applicable information regarding each topic.

### 2.5. Data Extraction

The selected studies were analyzed for relevant information to each PICO statement (Section 2.1). This information was recorded in an excel spreadsheet using the following columns:

1. Additives/Preservatives: Study ID, preservative type, exposure level, outcomes, effect direction, key findings, limitations, risk of bias, and quality grade.
2. Whole/processed Ingredients: Reference, ingredient, processing step, degree of processing, nutritional effects, digestibility effects, effect direction, outcome hierarchy, risk of bias, quality of evidence, and limitations.
3. Human-grade Nutrition Assessment: Reference, diet/ingredients, ingredient type, category, nutritional effects, digestibility effects, outcome hierarchy, effect direction, risk of bias, quality of evidence, limitations/key findings.
4. Human-grade Safety Assessment: Reference, ingredient type, outcome, measured level vs. human-grade regulatory limit, safety assessment, outcome hierarchy, effect direction, risk of bias, quality of evidence, and limitations/key findings.

### 2.6. Quality Assessment

After the data were extracted, each study was assigned a “Quality of evidence” grade on a scale of A, B, C, D, or F, which utilized aspects from the Cochrane GRADE workbook [40] to ensure a causal relationship was present. A quality grade of “A” contained randomization of subjects/inclusion, blinding to the inclusion, a measurable effect, absence of conflicts of interest/bias, and more than one dose of the inclusion to understand the dose–response relationship. If all details were present in the study, a quality grade of “A” was assigned, but with each missing detail, the grade dropped by one level. For instance, a study would receive a quality grade of “B” if it included randomization, blinding, measured food safety parameters, and multiple dosages, but had conflicts of interest—meaning a single methodological limitation resulted in a lower overall grade. The bias was assessed by analyzing the “conflicts of interest” section and thought patterns that did not address both sides of a concept. For example, a study that was funded by a company that could benefit from the findings or which did not address the negative aspects of a compound were deemed as “biased” and the quality of evidence was decreased by one letter grade.

#### 2.6.1. Risk of Bias Assessment

Each study was analyzed for factors that could contribute to study outcomes or study design. Aspects from the Cochrane Risk of Bias (RoB 2) tool were utilized to assess the potential for bias such as missing outcome data; bias in measurement of outcomes; source of funding; lack of proper randomization; and deviations from intended interventions. If any of the biases were detected, the study was classified as “High” likelihood, with a reduction in grade score. Studies with a lack of bias detected were classified as “Low”, with no effect on grade score. Each presence of a potential bias was weighed equally to ensure the consistency of the assessment.

#### 2.6.2. Finding Interpretations

Each study was assigned an effect direction classification based on the integrated assessment of quality grade, risk of bias evaluation, statistical outcomes, and reported biological significance. This classification system was utilized to synthesize heterogeneous outcomes across diverse study designs and facilitate systematic interpretation of findings.

For additives and preservatives, effect direction was determined by analyzing adverse event rates, toxicity indicators, and clinically relevant health outcomes. Studies demonstrating statistically significant increases in adverse events, toxicity markers, or negative health parameters were classified as “harmful”. Studies showing reductions in adverse events, improvements in health markers, or beneficial physiological responses were classified as “beneficial”. Studies with null findings, non-significant differences between treatment and control groups, or conflicting evidence across measured outcomes were classified as “neutral”.

For human-grade versus feed-grade ingredient comparisons, a dual classification approach was integrated to address both the safety and nutritional aspects. Safety assessments utilized regulatory thresholds established by the FDA and USDA for human food safety. Ingredients meeting or exceeding contaminant limits for mycotoxins, heavy metals, pathogens, or biogenic amines relative to human-grade standards were classified as “not suitable for human food”, while those below established thresholds were classified as “suitable for human food”. Nutritional assessments compared macronutrient compositions, micronutrient density, digestibility coefficients, and bioavailability between human-grade and feed-grade formulations, with outcomes reported as directional changes (increase or decrease) relative to the feed-grade baseline values.

For processing effects on ingredients, effect direction was established through analysis of digestibility coefficients, nutrient retention profiles, antinutrient reduction, and bioavailability metrics. Processing interventions resulting in improved digestibility, enhanced nutrient bioavailability, reduced antinutrient content, or increased antioxidant activity were classified as “beneficial”. Interventions which caused nutrient degradation, reduced digestibility, or decreased bioavailability were classified as “harmful”. Studies which reported no significant changes, mixed effects across multiple outcomes, or outcomes dependent on specific contextual variables were classified as “neutral”.

An outcome hierarchy classification was applied to studies examining human-grade ingredients and processing effects to establish the relative strength and applicability of evidence (Section 2.2.2 and Section 2.2.3). Primary outcomes included direct measurements and nutrient levels, contaminant concentrations, growth parameters, and metabolic indicators. Secondary outcomes represented digestibility and bioavailability assessments. Tertiary outcomes encompassed adverse reactions, performance metrics, antinutrient levels, and bioactive compound concentrations. This hierarchical structure allowed for prioritization of outcomes with the greatest clinical and nutritional relevance when synthesizing findings across heterogeneous studies, rather than classifying all outcomes as having equal impacts on overall pet health.

### 2.7. Processing of Ingredients Subgroup Analyses

In order to address the heterogeneity of processing methods across studies on whole or processed ingredients, a processing intensity classification system was deployed. These studies were categorized into three primary processing groups based on the degree of thermal, chemical, or mechanical modification.

#### 2.7.1. Minimal Processing: Methods Involving Limited Thermal Exposure and Mechanical Alteration

- Raw ingredients (no processing);
- Cold pressing (≤40 °C);
- Freeze-drying/lyophilization;
- Air-drying at ambient temperatures;
- Basic cleaning and washing.

#### 2.7.2. Moderate Processing: Methods Involving Controlled Thermal or Mechanical Treatment

- Steam cooking (≤100 °C);
- Light roasting (≤120 °C);
- Boiling/steaming (≤100 °C for ≤30 min);
- Germination and sprouting;
- Soaking and tempering;
- Coarse milling and grinding.

#### 2.7.3. Intensive Processing: Methods Involving High Thermal, Mechanical, and/or Chemical Treatment

- Extrusion (>120 °C, high pressure);
- High-temperature roasting (>120 °C);
- Pressure cooking (>100 °C, high pressure);
- Fine milling and mechanical disruption;
- Chemical preservation and pH modification;
- Retort sterilization.

#### 2.7.4. Effect Direction Categorization

- Beneficial effects: Improved digestibility, increased antioxidants, and reduced antinutrients (overall net positive).
- Neutral effects: No significant changes or mixed effects (harmful + beneficial).
- Harmful effects: Nutrient losses and reduced digestibility or bioavailability (overall net negative).
- For processing analysis, grade A studies were allotted 3 points per effect direction, 2 points were allotted for grade B, and 1 point for grade C.

## 3. Additives and Preservatives Are Harmful to Pets

### 3.1. Additives vs. Preservatives

**Figure 2:**
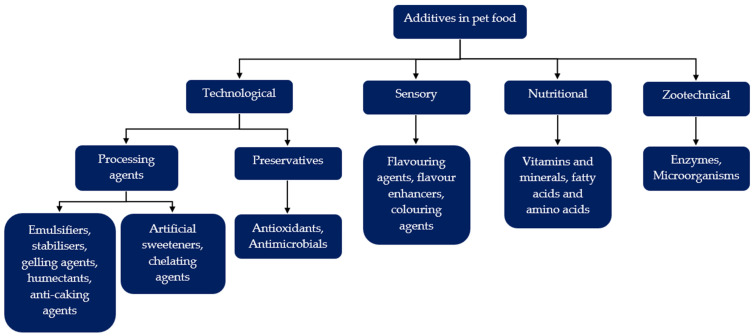
Additives in pet food.

This category is broad, as many ingredients can be classified as additives, but this review will distinguish between additives and preservatives because many claims consistently mentioned both. Additives are defined by AAFCO as “An ingredient or combination of ingredients added to the basic feed mix or parts thereof to fulfill a specific need”. It is usually used in microquantities and requires careful handling and mixing [41]. The AAFCO definition of preservatives is “A substance added to protect, prevent, or retard decay, discoloration, or spoilage under conditions of use or spoilage” [41]. The flow chart in Figure 2 depicts the relationship between additives and preservatives within the context of pet food [42].

### 3.2. Systematic Review of Additives in Pet Food

**Figure 3:**
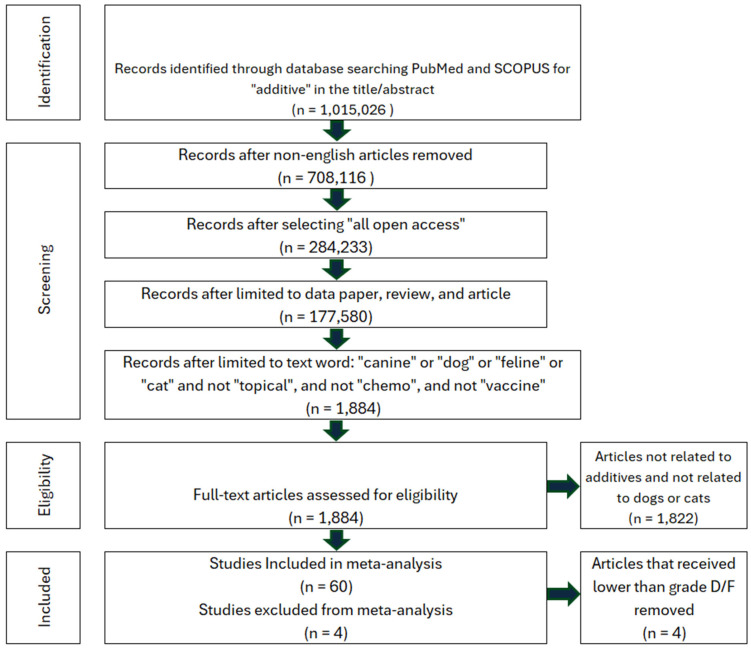
PRISMA flow diagram showing selection criteria and screening process for additive studies. Note. The flowchart illustrates the systematic review methodology used to identify relevant studies on pet food additives, showing identification, screening, eligibility assessment, and final inclusion phases.

The number of additives in pet food is vast and the scientific basis for these determinations may be insufficient for ensuring long-term safety [42]. To properly assess the safety of additives, a systematic review was performed on all available literature using the search criteria in Figure 3. The studies were assessed for additives that were deemed harmful to either dogs or cats. Each eligible study was reviewed for acute toxicity, chronic toxicity, or adverse reactions (diarrhea, vomiting, aggression, lethargy, weakness, etc.) which can be seen in Table 1.

### 3.3. Systematic Review of Preservatives in Pet Food

**Figure 4:**
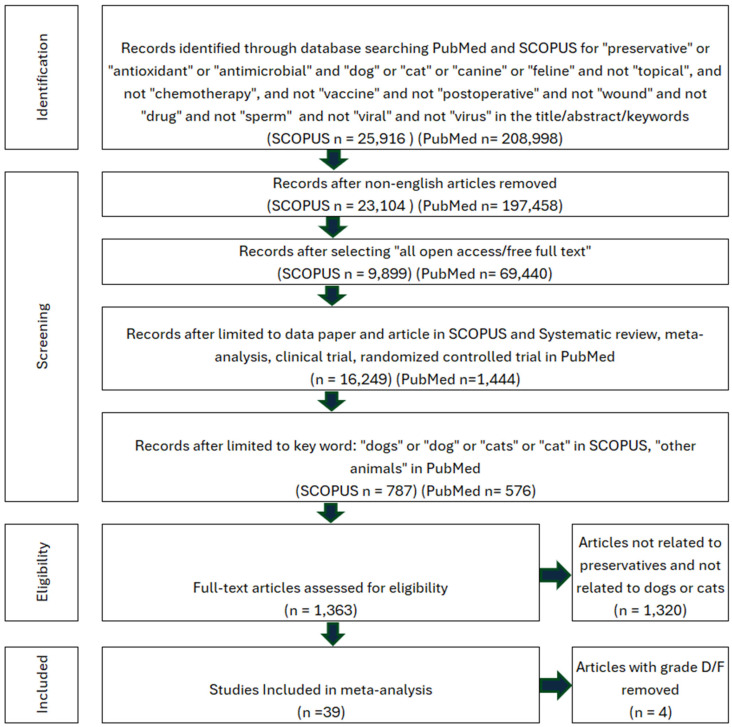
PRISMA flow diagram showing selection criteria and screening process for preservative studies. Note. The flowchart illustrates the systematic review methodology used to identify relevant studies on pet food preservatives, showing identification, screening, eligibility assessment, and final inclusion phases.

Preservatives are added to pet foods in order to prevent oxidative rancidity, microbial growth, and degradation of essential nutrients. However, a study found that food containing preservatives received one of the most negative ratings from consumers, with an average score below 3 on a 1–7 scale (M = 2.235, SD = 1.348) [105]. To understand this phenomenon, a systematic review was performed using the search criteria in Figure 4. The studies were assessed for preservatives that were deemed harmful to either dogs or cats. Each eligible study was reviewed for acute toxicity, chronic toxicity, or adverse reactions (diarrhea, vomiting, aggression, lethargy, weakness, etc.) which can be seen in Table 2.

## 4. Human-Grade Ingredients Are Safer and More Nutritious

**Figure 5:**
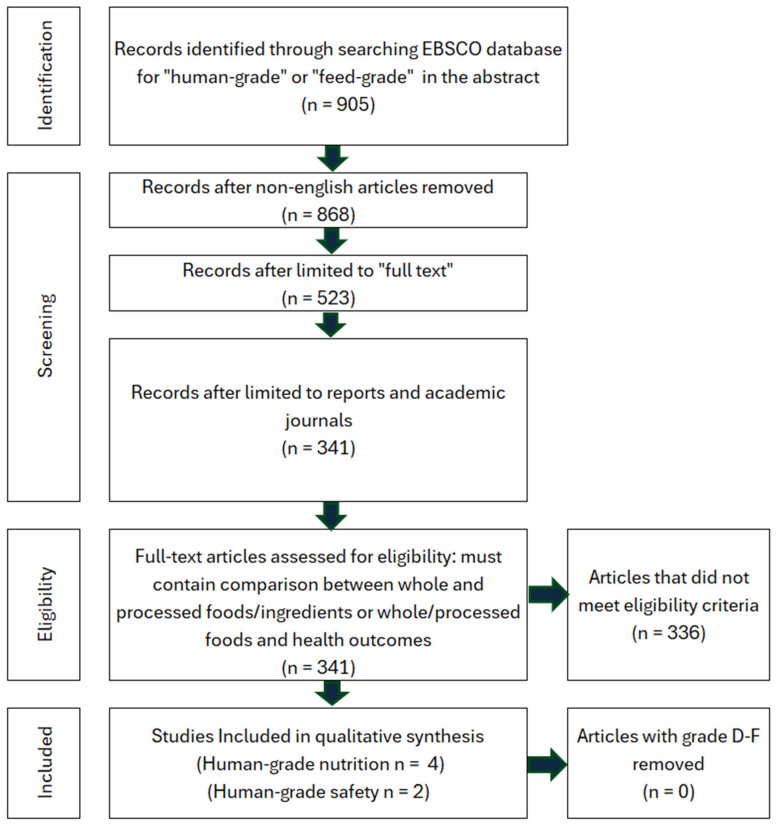
PRISMA flow diagram showing selection criteria and screening process for human-grade vs. feed-grade studies. Note. The flowchart illustrates the systematic review methodology used to identify relevant studies on human and feed-grade ingredients showing identification, screening, eligibility assessment, and final inclusion phases.

The utilization of human-grade ingredients aligns with the humanization of pet food, which is occurring alongside the increase in pet ownership [147]. Human-grade ingredients are produced, handled, and processed in the United States to meet stringent standards set by the Food and Drug Administration (FDA) and U.S. Department of Agriculture (USDA) which mandate specific guidelines for cleanliness, contamination limits, traceability, and processing conditions. This also means that each ingredient in a recipe must be classified as human-grade to meet human consumption standards. Feed-grade ingredients are regulated differently by the AAFCO and FDA, which includes different standards for nutritional composition, contaminant limits, labeling, good manufacturing practices, and regulatory oversight. Human-grade ingredients may offer a higher standard of safety [148], enhanced nutritional value [149], and improved gut health [149,150]. Feed-grade ingredients are not suitable for human consumption due to the inclusion of byproducts and parts from animals that are classified as “dying, disabled, diseased, or deceased [151]”. The search criteria to retrieve human-grade and feed-grade studies can be seen in Figure 5. Study-level assessment for nutritional aspects can be seen in Table 3a, while the safety assessment can be seen in Table 3b.

## 5. Whole Ingredients Are Healthier than Processed Ingredients

**Figure 6:**
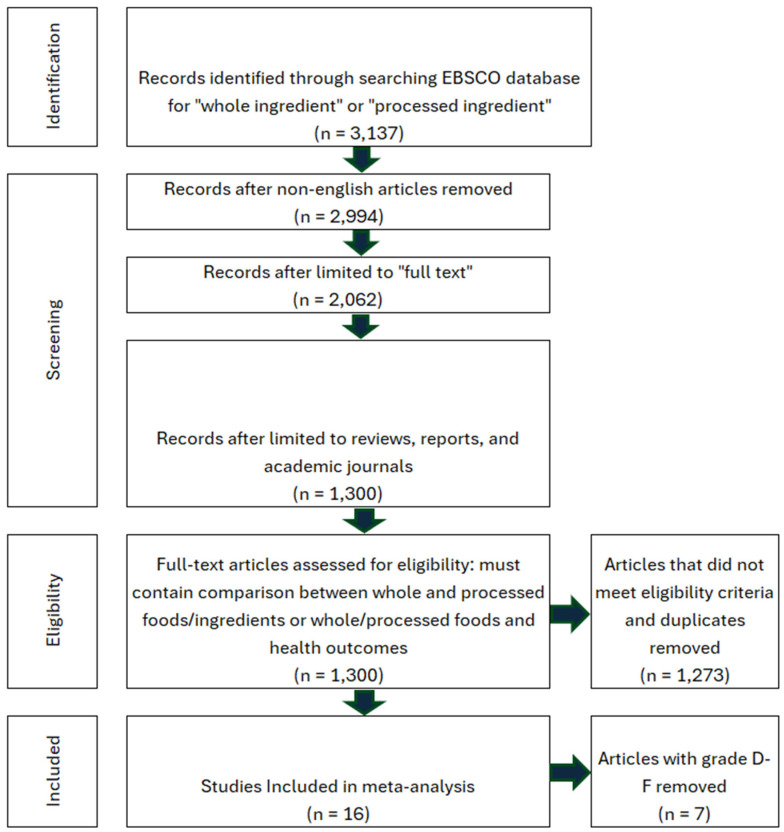
PRISMA flow diagram showing selection criteria and screening process for whole vs. processed ingredient studies. Note. The flowchart illustrates the systematic review methodology used to identify relevant studies on pet food ingredient processing, showing identification, screening, eligibility assessment, and final inclusion phases.

Currently, there is no specific organization that governs “whole ingredient” claims for pet food. Pet food manufacturers would need to ensure that their products are aligned with FDA and State Department of Agriculture regulations, which are typically adopted from AAFCO standards [155,156]. The AAFCO requires “whole grain” claims to be listed in their whole form on the ingredient label such as brown rice or whole wheat. The ingredient must follow “AAFCO officially defined animal feed ingredient” terminology and “No reference to quality or grade of ingredients can be made in the ingredient statement” [157,158]. However, processing these grains to produce pet food yields unrecognizable grains due to the nature of heat, milling, emulsification, and extrusion. This means that consumers have little confirmation that pet foods are supplied with whole ingredients that have not been modified via processing. Alternatively, human-grade pet food diets which claim to utilize whole grains are required to contain at least 8 g of dry, whole grain ingredients per reference amount customarily consumed (RACC) [159]. The FDA also requires that whole grain ingredients contain all parts of the grain (bran, germ, and endosperm) in their natural proportions [160]. Figure 6 provides the search criteria utilized to gather whole vs. processed ingredient studies. The study-level assessment was conducted and displayed in Table 4.

## 6. Results and Discussion

### 6.1. Pet Food Additives

#### 6.1.1. Study Selection and Characteristics: Additives

**Figure 7:**
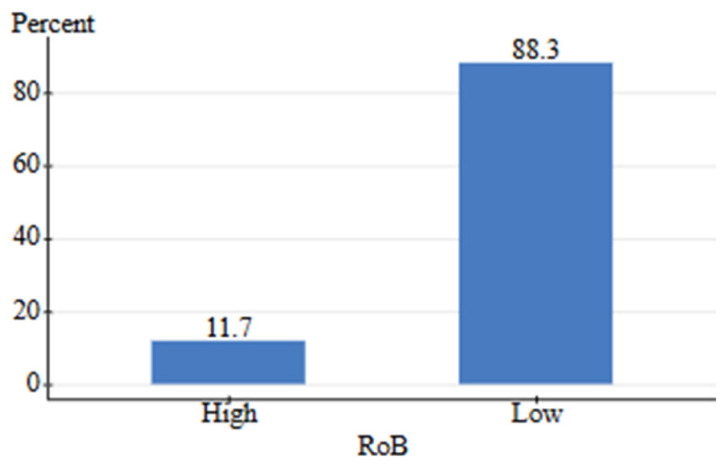
Risk of bias: additives.

The systematic literature search identified 64 studies examining the effects of pet food additives (zootechnical, nutritional, and functional ingredients) on adverse health outcomes. A total of 60 studies met inclusion criteria and had calculable effect sizes, allowing for complete data utilization without further exclusions (Figure 3). Four studies were removed as these were in vitro or review articles with no extractable data. Risk of bias assessment resulted in a relative frequency of 11.7%, with 7 studies showing high potential for bias (Figure 7).

The 60 included studies contained 33 dog studies (55%) and 27 cat studies (45%), a total of 3218 participants, with 1304 controls, 1914 undergoing treatment (mean = 53 participants). Study durations ranged from 4 to 560 days (mean = 69.6 days, interquartile range = 52.5 days). Additive types included preservatives, probiotics, antioxidants, enzymes, and other functional ingredients. The quality assessment yielded 17 grade A studies (28.3%), 33 grade B studies (55%), and 10 grade C studies (16.7%). Approximately 8 out of 60 studies reported any events, with 86.7% of studies reporting no adverse events in either control or additive groups. The adverse event rate was 2.3% amongst control groups while additive groups had a 2.66% adverse event rate. A primary analysis consisted of a Bayesian risk difference meta-analysis with moderators, Bayesian meta-regression, and a supplemental Peto meta-analysis to estimate heterogeneity without continuity corrections.

#### 6.1.2. Primary Bayesian Meta-Analysis with Risk Difference Effect Size: Additives

**Figure 8:**
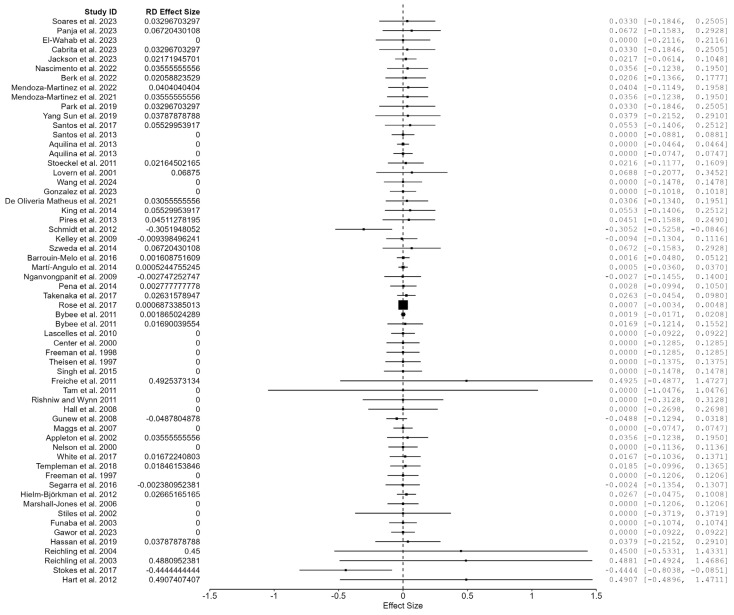
Forest plot for additives. Note. Citations for each comparison are listed in the left most column under “Study ID” [43,44,45,46,47,48,49,50,51,52,53,54,55,56,57,58,59,60,61,62,63,64,65,66,67,68,69,70,71,72,73,74,75,76,77,78,79,80,81,82,83,84,85,86,87,88,89,90,91,92,93,94,95,96,97,98,99,100].

The risk difference effects sizes were calculated using adverse event totals for the additive and control groups in each of the 60 studies. Bayesian methodology revealed a pooled risk difference (RD) estimate of 0.0006 (95% CI: −0.190, 0.892), translating to a 0.006% increase in adverse events for additive groups (Table 5). While not statistically significant, the estimation implies a slight increase in adverse event potential in the additive group when compared to the control. This aligns with a 0.36% increase in additive adverse event rates (2.66% vs. 2.3% control). Alongside preservatives, the confidence interval crossed the null value of 0, indicating that the effect was not statistically significant at 95% CI. The BF_10_ showed a null effect of 0.055, providing strong evidence against the presence of meaningful harm and translating to 18:1 odds in favor of the null hypothesis. A sensitivity analysis was performed by excluding “High” RoB studies to understand the effects of bias on estimations, leading to a near-identical BF_10_ = 0.057 (95% CI: 0.447, 0.508). This finding shows a lack of impact from low-quality studies on overall effect size estimates and indicates robustness (Figure 8).

#### 6.1.3. Moderation in Bayesian Risk Difference Meta-Analysis: Additives

Moderation was performed using the Bayesian risk difference model with species listed as subgroups and predictors such as quality grade, duration length, additive type, and RoB. Results displayed weak evidence for effect modifications, with Bayes factors consistently below 1.0 (Table 6). Species differences existed, as cats (BF_10_ = 0.396) showed slightly weaker evidence against moderation when compared to dogs (BF_10_ = 0.119). Duration length provided the strongest evidence for moderation in dogs (BF_10_ = 0.066), with virtually no effect from exposure time to additives. Results for cats showed weaker evidence (BF_10_ = 0.490) but still favored no moderation by duration length. Consistent with previous moderators, quality grade showed weak evidence for inclusion with cats (BF = 0.4410) showing weaker evidence against moderation than dogs (BF = 0.117). The same trend was observed when moderating by risk of bias, implying that methodological quality did not have a significant impact on the null findings (cats BF = 0.552, dogs BF = 0.158). Across all moderator assessments, inclusion Bayes factors ranged from 0.066 to 0.552, meaning moderate evidence was found against the inclusion of moderators. This consistent pattern supports the null findings and confirms the homogeneous effects that additives may have on pet food.

**Figure 9:**
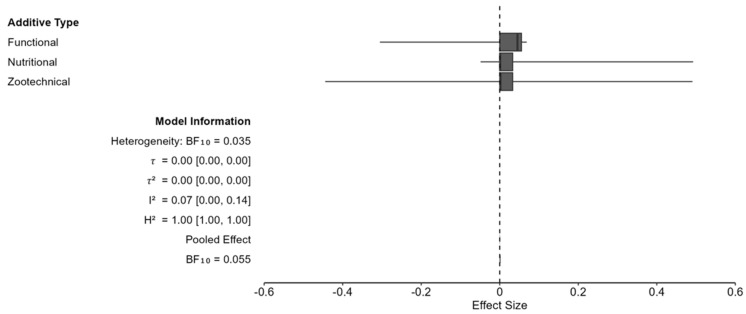
Forest plot by additive type.

When moderating by additive types such as functional, nutritional, and zootechnical ingredients, the effect sizes portray a cluster located at 0.05. The consistency of the effects is evident, but none reached statistical significance, with all ranges including the null value of 0. The functional additive type provides an opposing effect direction from the nutritional additives, while the zootechnical show bidirectional effects (Figure 9). More research is needed in this area to confirm true effect sizes for each additive type, informing consumers of true risk differences. While not significant, the effect direction is null, and additives appear to have no effect on pet adverse event rates.

#### 6.1.4. Heterogeneity: Classical Peto Meta-Analyses Additives

In order to obtain an accurate assessment of heterogeneity between studies and impact on effect size estimates, a Peto meta-analysis was selected for the ability to handle large quantities of double-zero studies and the observed heterogeneity. Results confirmed this observed heterogeneity with *I*^2^ = 73.31% and H^2^ = 3.747, with a statistically significant meta-analytic test *Q_e_*(59) = 18.73 (*p* = 0.002). The Bayesian analysis methodology was able to successfully handle this heterogeneity with an *I*^2^ = 0.068% (95% CrI: 0.000 to 0.138) and H^2^ = 1.001 ((95% CrI: 1.000 to 1.001). The BF_10_: 0.035 indicates 29:1 odds against the presence of heterogeneity by focusing heavily on prior distribution for *τ*, which was <0.01, driving the estimate towards 0. The Peto model provides a stronger heterogeneity estimate as it contains model-free, objective variance decomposition without requiring prior information.

#### 6.1.5. Publication Bias Assessment: Additives

**Figure 10:**
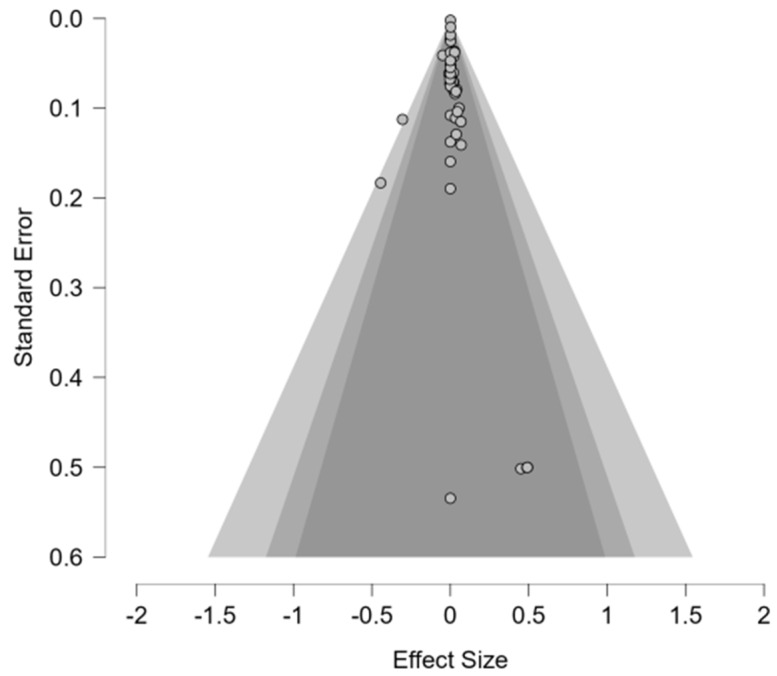
Funnel plot for additives. Note. Each point represents a single study, plotted by its effect size on the horizontal axis and its standard error on the vertical axis. The shaded regions indicate the areas where studies would be expected to lie in the absence of publication bias, with lighter shading representing larger expected effects at higher standard errors.

Unlike the Publication Bias Assessment for preservatives, the regression tests yielded converging results. The meta-regression test, *z* = 0.927, (*p* = 0.354), and weighted regression test, *t*(58) = 1.500, (*p* = 0.139), both provided evidence against significant asymmetry, providing reassurance in the effect estimates. Fail-safe N analysis showed values of 0 across the Rosenthall, General, and Rosenburg tests, while the Orwin test showed that 60 studies are needed to nullify the findings from this study. Overall, the combination of tests was suggestive of low risk of publication bias, and visual inspection of Figure 10 aligns with these results.

#### 6.1.6. Bayesian Model Diagnostics and Convergence: Additives

Upon MCMC convergence assessment, the values delivered strong convergence across all 60 studies with multiple moderators. R-hat values ranged from 1.001 to 1.013, demonstrating strong convergence. Effective sample size values for cats ranged from 8883 to 38,624, while for dogs, they ranged from 1269 to 23,041, indicating higher chain mixing. The wider posterior distributions provide a more efficient exploration of parameter space and MCMC sampling with minimal autocorrelation. MCMC error ranged from 0.005 to 0.011 for cat parameters and 0.007 to 0.028 for dog parameters. MCMC errors consistently below 3% of the posterior standard deviation are indicative of true posterior uncertainty rather than simulation noise. Overall, model convergence was excellent, and ESS metrics provided evidence for well-mixed chains. One exception was zootechnical additives for dogs, which contained a marginally high R-hat = 1.013 and should be further investigated in future research.

### 6.2. Bayesian Meta-Analysis: Pet Food Preservatives

#### 6.2.1. Study Selection and Characteristics

**Figure 11:**
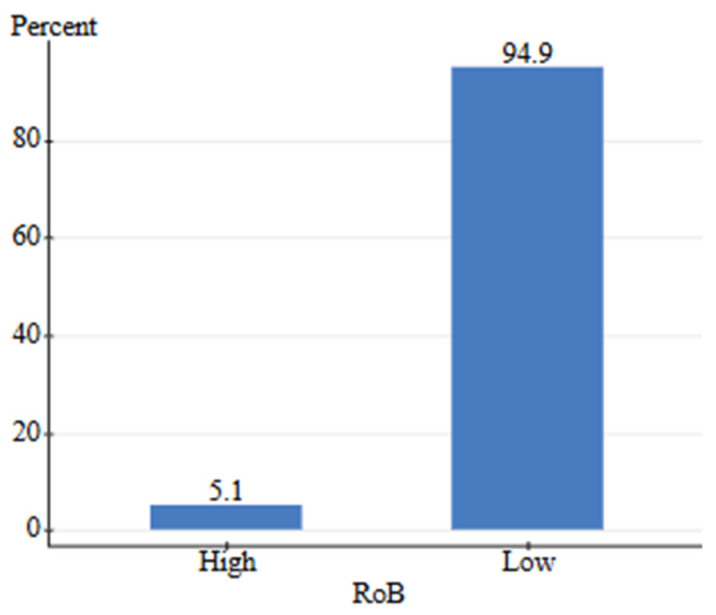
Risk of bias: preservatives.

**Figure 12:**
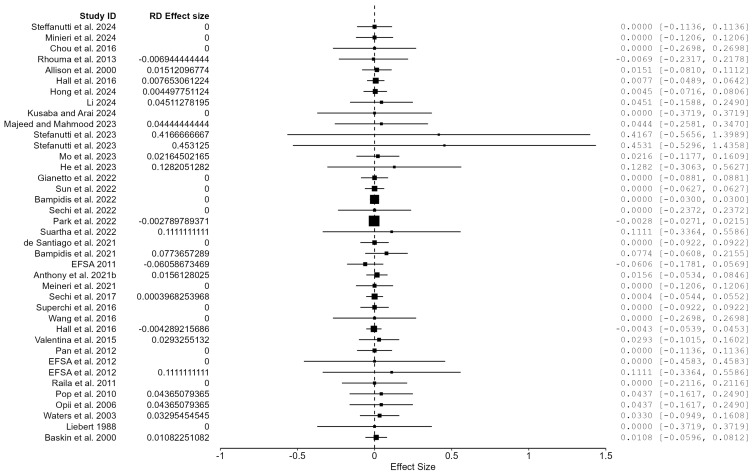
Forest plot: preservatives. Note. Citations for each comparison are listed in the left most column under “Study ID” [106,107,108,109,110,111,112,113,114,115,116,117,118,119,120,121,122,123,124,125,126,127,128,129,130,131,132,133,134,135,136,137,138,139,140,141,142].

The systematic literature search identified 43 studies meeting predefined inclusion criteria. Four studies were excluded as they did not contain treatment and control groups with extractable data [134,135,136,137]. The risk of bias assessment yielded a relative frequency of 5.1% for high potential of bias within included studies (Figure 11). Of these, 39 studies with calculable effect sizes were included in the final Bayesian meta-analysis (Figure 12).

The 39 included studies comprised 30 dog studies (77%) and 9 cat studies (23%), with a total of 1613 participants (644 control, 969 treatment). Study durations ranged from 14 to 941 days (median = 60 days, interquartile range = 28–168 days). Grade A studies comprised 13% of studies, with 66% grade B studies and 20% grade C studies. Study duration tended to be longer rather than shorter, with 77% of studies measuring preservatives impact >30 days. Studies with dogs as the primary species accounted for 77% of the dataset, with cats accounting for 23% of dataset. The adverse event rate was approximately 0.15% in the control group and 1% in the preservative group.

#### 6.2.2. Primary Bayesian Meta-Analysis with Risk Difference Effect Size: Preservatives

Effects size computations were calculated using adverse event totals for the preservative and control groups in each of the 39 studies. Bayesian methodology revealed a pooled risk difference (RD) estimate of 0.0003 (95% CI: −0.190, 0.892), corresponding to a 0.03% increase in adverse events for preservative groups (Table 7). While not statistically significant, this estimate suggests that preservatives provide a slight increase in adverse reactions in comparison to the control, matching the 0.85% increase in preservative adverse event rates (0.15% vs. 1% control). However, the confidence interval included the null value of 0, indicating that the effect was not statistically significant at 95% CI. Likewise, these results translate into 6:1 odds in favor of the null hypothesis of no effect for preservatives. The Bayes factor (BF) showed a null effect of 0.162. A sensitivity analysis was conducted to understand the effects of high RoB studies on RD, resulting in 2 excluded studies, with 37 remaining in the analysis. The estimate for RD = 0.0004 (95% CI: −0.004, 0.009) was consistent with the all-studies analysis and was not statistically significant. This result warranted no further subgroup analysis to examine the data for bias as the difference was negligible.

#### 6.2.3. Moderation in Bayesian Risk Difference Meta-Analysis: Preservatives

In order to assess the effect of moderators on risk difference effect size estimates, Bayesian meta-analysis and meta-regression were performed. Species was utilized as a subgroup, with duration length, study quality, RoB, and preservative type assigned as predictors. This yielded weak evidence against the moderation of species, preservative type, duration, quality grade, and RoB (Table 8). Each moderated BF stayed below 0.601, suggesting that no evidence is present that the studied characteristics modify preservative adverse events. Notably, cats exhibited greater uncertainty from higher BF estimates and wider confidence intervals.

**Figure 13:**
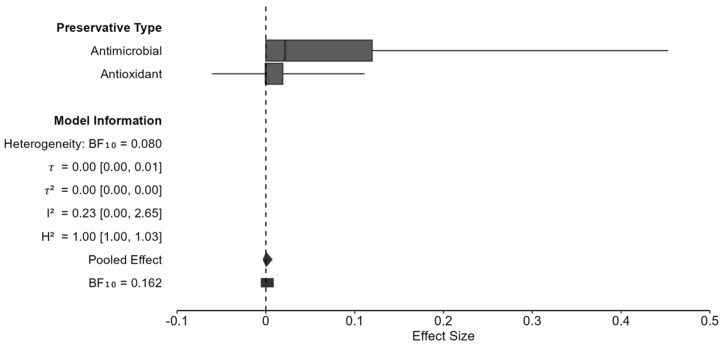
Forest plot by preservative type.

Moderation by preservative type showed clear forest plot differences in Figure 13 as antimicrobial preservatives showed greater variability in RD effect sizes and more adverse events when compared to antioxidants. Likewise, the effect direction was slightly negative and warrants further research to elucidate the potential correlations between antimicrobial preservatives and negative effects in pets.

#### 6.2.4. Heterogeneity: Classical Peto Meta-Analysis on Preservatives

In order to understand the heterogeneity of the included studies without continuity corrections or exclusion of double-zero studies, a Peto meta-analysis was conducted using risk difference effect sizes. The results yielded an *I*^2^ = 75.45%, H^2^ = 4.704, and *Q_e_*(38) = 4.07 (*p* = 0.044), proving the presence of statistically significant between-study heterogeneity. On the contrary, Bayesian averaging was able to handle this efficiently with an *I*^2^ = 0.23 (95% CrI: 0.000 to 2.646) and *τ* = 4.674 × 10^−4^ (95% CrI: 0.000 to 0.007), indicating most of the posterior probability mass is located at zero heterogeneity (Table 7). BF = 0.08 also provided strong evidence against heterogeneity, likely due to the 0.5 continuity corrections in computing the RD effect size. The authors observed large amounts of heterogeneity in study design, populations, dosing, and outcomes, which aligned more so with the Peto estimate for heterogeneity rather than the Bayesian estimate. When moderating by species, both dogs and cats showed Bayes factors which strongly favored the null model of zero heterogeneity. In other words, the inclusion of dogs (BF = 0.093) and cats (BF = 0.238) showed 11:1 and 4:1 odds against the presence of heterogeneity.

#### 6.2.5. Publication Bias Assessment: Preservatives

**Figure 14:**
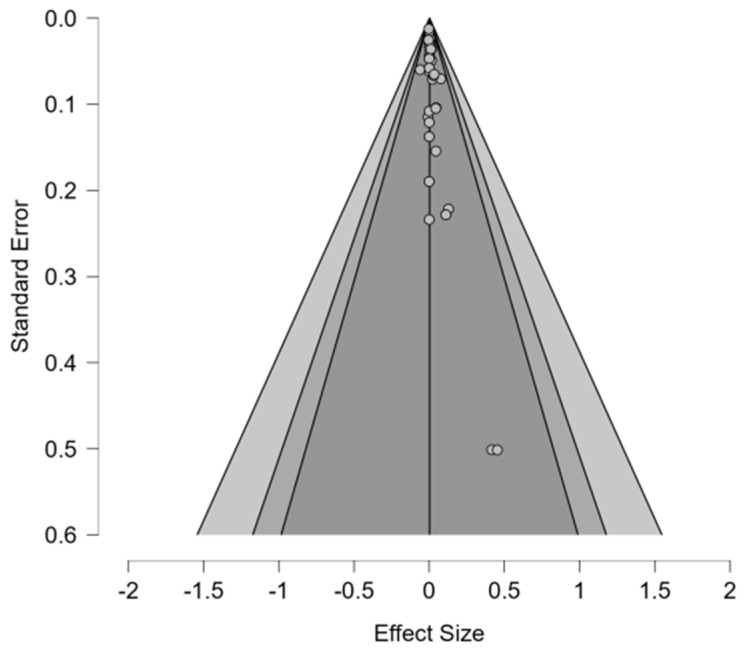
Funnel plot: preservatives. Note. Each point represents a single study, plotted by its effect size on the horizontal axis and its standard error on the vertical axis. The shaded regions indicate the areas where studies would be expected to lie in the absence of publication bias, with lighter shading representing larger expected effects at higher standard errors.

Publication bias was assessed via a random-effects methodology with fixed effects under H1, utilizing Bayesian risk difference estimates for each of the 39 studies. Results show no clear signs of asymmetry within the funnel plot (Figure 14). However, regression tests showed conflicting results, as a meta-regression test, *z* = 1.307 (*p* = 0.191), suggested non-significant asymmetry, while a weighted regression test, *t*(37) = 3.789 (*p* < 0.001), found highly significant asymmetry. The discrepancy between tests can result from smaller studies with null findings or negative findings that are underrepresented. All fail-safe N metrics (Rosenthal, General, and Rosenburg) delivered values of 0 besides Orwin = 39, indicating that 0–39 unpublished studies are required to nullify the observed effects. This result aligns with the null pooled estimate and implies that the meta-analysis is robust. Overall, the combination of conflicting asymmetry tests and zero fail-safe N metrics indicates patterns of potential publication bias which require assessment in future research.

#### 6.2.6. Bayesian Model Diagnostics and Convergence

Bayesian MCMC assessment delivered excellent convergence across both species as subgroups. All R-hat values were ≤1.001 with MCMC error ranges = 0.005–0.010 (<0.05). The effective sample size (ESS) ranged from 10,682 to 33,867, well beyond the recommended 1000 for stable estimates. Estimates for dogs showed ESS = 10,682, R-hat = 1.001, and MCMC error = 0.01. Estimates in cats showed elevated ESS = 15,099, R-hat = 1.0, and MCMC error = 0.006. This assessment indicates that MCMC chains properly explored the posterior distribution without the need for autocorrelation and ensures that estimates are precise, not dominated by simulation noise.

### 6.3. Meta-Analysis and Meta-Regression: Effect of Processing on Digestion

#### 6.3.1. Dataset Overview and Scope: Impact of Processing on Digestion

**Figure 15:**
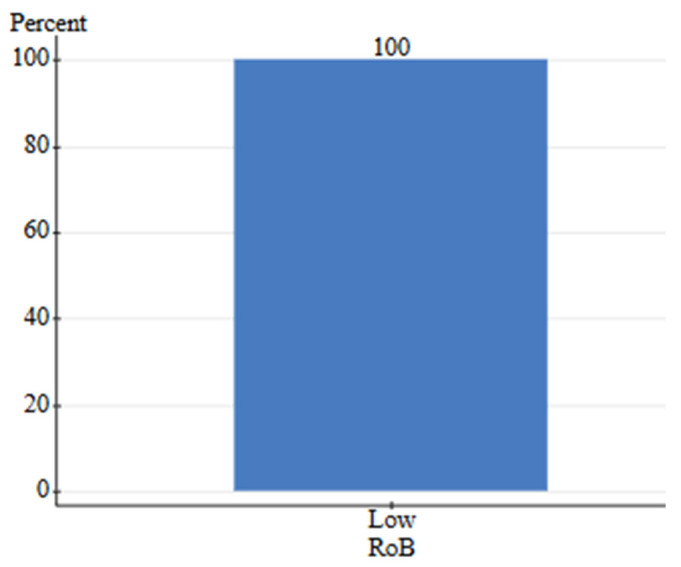
Risk of bias assessment: digestion.

A total of five high-quality studies (all grade A) containing 102 effect size comparisons were included in the random-effects meta-analysis, which aimed to quantify the impact of processing on digestibility. This yielded 15 different ingredients across 18 different processing methods, covering 16 distinct outcome categories such as protein, starch, and total tract/ileal digestibility. The studies contained 62.7% intensive, 19.6% intensive/moderate, 16.7% moderate, and 1% minimal degree of processing. Risk of bias assessment showed a relative frequency of 0% for studies with high potential bias (Figure 15).

#### 6.3.2. Overall Effect of Processing on Digestion

**Figure 16:**
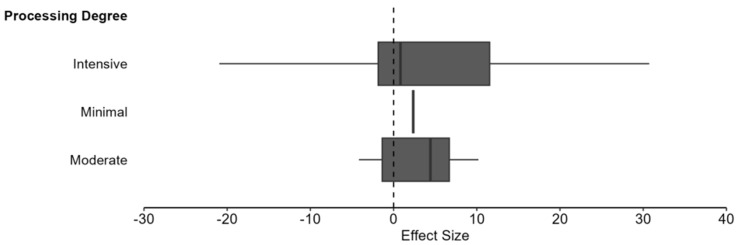
Forest plot of processing intensity on digestibility. Effect size values beyond 0 represent a positive or negative effect based on the placement on the x-axis. A positive effect size represents a beneficial effect from processing degree, while negative effect sizes indicate a harmful effect from processing degree. The solid-black vertical lines are located at the mean for each processing degree and horizontal solid lines represent the 95% CI.

Processing delivered mixed effects, with predominantly harmful impacts on digestibility. In 50% of comparisons, digestibility was reduced, while 27.5% showed improvements in digestibility and 22.5% showed no significant change. The effect size magnitude was large, as 88.2% of effects caused substantial changes (Standard Mean Difference > 0.8). Likewise, the pooled Standardized Mean Difference (SMD) was 1.971 (95% CI: 0.567, 3.374, *p* = 0.005). This result indicates that, across all processing methods, intensities, and ingredients, processing generally improves the digestibility of ingredients by approximately two standard deviations when compared to unprocessed controls. The mean percent change across studies was +51.3%, with direction varying depending on outcome type. A clear dose–response relationship emerged, with increasing processing intensity associated with progressively larger digestibility changes (minimal: +7.8%, moderate: +27.7%, intensive: +75.1%). These differences in processing intensity can be seen in Figure 16.

#### 6.3.3. Meta-Regression: Processing Intensity on Digestibility

**Figure 17:**
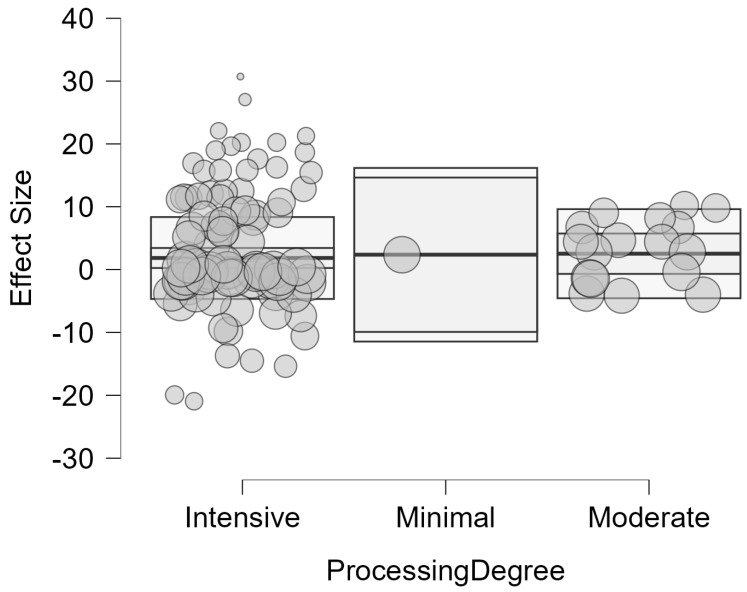
Bubble plots: impact of processing on digestion. Each point represents the effect size from a single comparison, grouped by the degree of processing (intensive, minimal, moderate). The boxes show the median and interquartile range of effect sizes within each processing category, while whiskers indicate the range of values, summarizing the distribution of effects for each level of processing.

The impact of processing intensity on digestibility of ingredients was substantial and statistically significant across the 102 study comparisons. Results showed a pooled SMD = 1.8 (95% CI: 0.139 to 3.462, *p* = 0.034) for intensive degree of processing, indicating a significantly positive impact on digestibility. Moderate degree of processing showed a clear improvement in digestibility in comparison to intensive methods SMD = 2.631 (95% CI: 0.162 to 5.100, *p* = 0.038), while the impact of minimal methods could not be calculated due to insufficient data (k < 2). Heterogeneity comparisons between intensive (*I*^2^ = 97.66%) and moderate (*I*^2^ = 83.25%) methods revealed more consistency in the results for methods with a moderate degree of processing (Figure 17). However, the degree of processing was not a significant moderator of digestibility outcomes and did not explain the large heterogeneity (QM(1) = 0.34, *p* = 0.562).

#### 6.3.4. Meta-Regression: Ingredient Type on Digestibility

When moderating by the type of ingredient, the strongest contributor to effect size heterogeneity was discovered (QM(9) = 59.99, *p* < 0.001). Huge variability was observed between ingredient type effect sizes, suggesting the importance of specifying an ingredient matrix prior to assessing the risk of processing intensity or method. Navy beans demonstrated a large beneficial effect (SMD = 14.095, 95% CI: −69.595, 97.785), alongside yellow peas (SMD = 9.074, 95% CI: −11.909, 30.057) and resistant starches (SMD = 6.10, 95% CI: −0.155, 12.356). Ingredients such as chickpeas (SMD = 0.463, 95% CI: −40.462, 41.388), beef byproducts (SMD = −0.722, 95% CI: −1.256, −0.188), and poultry byproducts (−0.376, 95% CI: −1.013, 0.262) showed null impacts in digestibility. Conversely, whole rice (SMD = −1.325, 95% CI: −4.712, 2.063) demonstrated negative effects from moderate/intensive processing on protein digestibility (Table 9). Overall, ingredient type was responsible for the largest impacts on digestion across all processing methods and intensities. As such, processing recommendations should be tailored to the recipe rather than determined by interpreting the effect size alone.

#### 6.3.5. Meta-Regression: Processing Method on Digestibility

Moderation by processing method yielded strong statistical associations with digestibility outcomes (QM(8) = 38.24, *p* < 0.001). This means that the method of processing is substantially more important than the degree of processing within that given method. For instance, selecting roasting or extrusion is more explanatory of digestibility outcomes rather than the temperature, pressure, or time used within these processes. Controlled thermal processing revealed a potential dose–response pattern within temperature. At 0.5 h duration, 95 °C produced an SMD = 2.572 (*p* = 0.058), 120 °C produced an SMD = 5.116 (*p* = 0.070), and 140 °C produced an SMD = 7.404 (*p* = 0.108). Likewise, boiling (SMD = 4.160, 95% CI: 0.382, 7.938) and wet roasting + tempering (SMD = 6.514, 95% CI: −5.313, 18.341) produced largely beneficial outcomes on digestion. Significant variability exists between processing methods, warranting future research to identify the optimal methods for digestion.

#### 6.3.6. Heterogeneity and Variability: Processing on Digestion

Due to high heterogeneity (*I*^2^ = 87.326%, *τ*^2^ = 10.065, *Q*(101) = 796.91, *p* < 0.001) across digestibility studies, a meta-regression was necessary to understand the contributing factors to the effect size. These heterogeneity values indicate that 87% of observed variation in effect size originates from true between-study differences, with observed variance 10x greater than what is expected from sampling error alone. The wide prediction interval (−4.477 to 8.419) suggests that effects could range from highly negative to highly positive impacts on ingredient digestibility. As expected, the effects of processing appear to be highly context-dependent. This can likely be explained by different outcome types (protein, starch, and total digestibility), ingredient types (legumes, grains, and animal byproducts), and processing method parameters (time, temperature, and moisture).

#### 6.3.7. Publication Bias: Impact of Processing on Digestion

**Figure 18:**
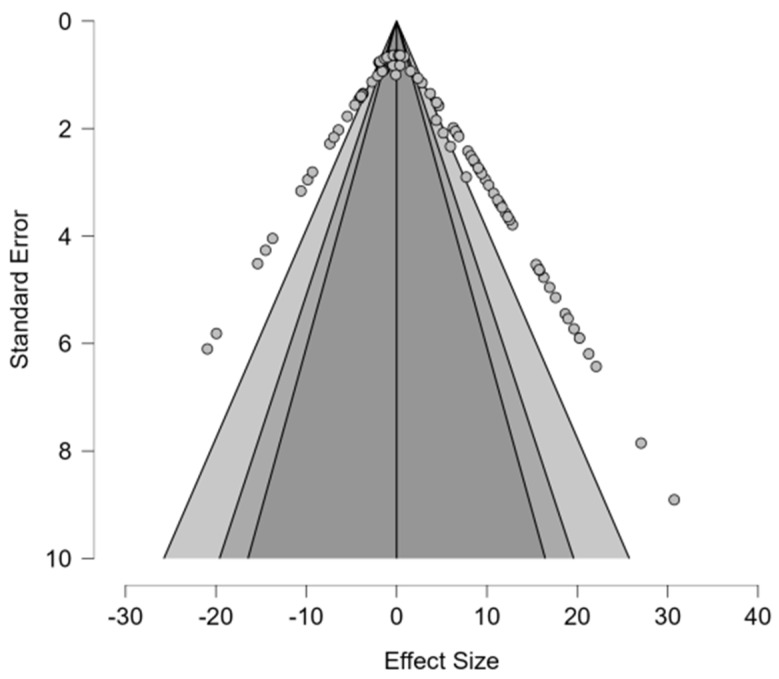
Funnel plot: impact of processing on digestion. Note. Each point represents a single study, plotted by its effect size on the horizontal axis and its standard error on the vertical axis. The shaded regions indicate the areas where studies would be expected to lie in the absence of publication bias, with lighter shading representing larger expected effects at higher standard errors.

Weighted regression and meta-regression testing showed significant publication bias (*t*(100) = 5.681, *p* < 0.001, *z*(100) = 10.56, *p* = <0.001). These test statistics indicate that smaller studies with null or positive effects are underrepresented in the published literature (Table 10). Likewise, the asymmetry estimate (μ = −2.393, 95% CI: −3.392 to −1.394) indicates that smaller studies report systematically larger beneficial effects, potentially stemming from methodological differences between smaller studies (Figure 18). Fail-safe N testing counteracted the publication bias with an extremely high Rosenthal fail-safe N = 2107 and Orwin fail-safe N = 102. Likely, the true effect size is less positive than the estimate provided and requires future research to elucidate.

### 6.4. Meta-Analysis and Meta-Regression: Effects of Processing on Nutrient Content

#### 6.4.1. Dataset Overview and Scope: Nutrients

**Figure 19:**
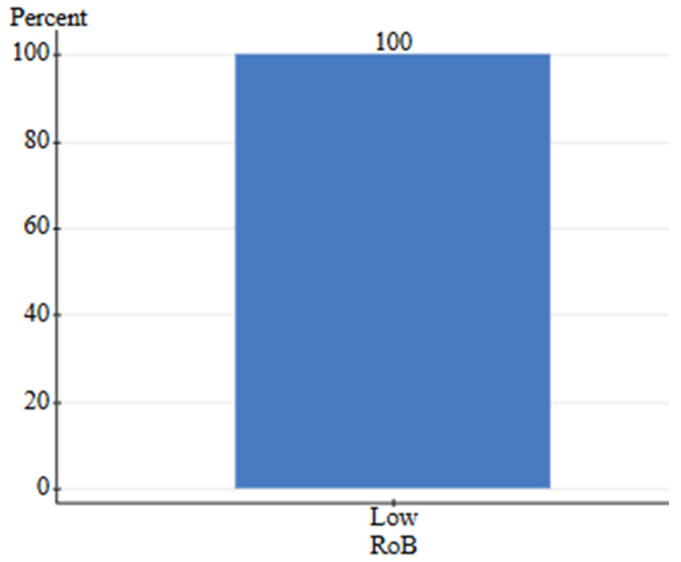
Risk of bias: impact of processing on nutrients.

A total of 137 effect size comparisons were extracted from six studies, 69.7% grade A studies and 30.3% grade C, respectively. This analysis contained 25 different ingredients across 19 different processing methods, yielding 23 distinct outcomes across 5 nutrient categories. These factors contributed to a more heterogeneous dataset with more beneficial effects overall (61.1% vs. 27.5% in digestion). The risk of bias assessment showed a relative frequency of 0% high potential bias studies (Figure 19).

#### 6.4.2. Overall Effects of Processing on Nutrients

**Figure 20:**
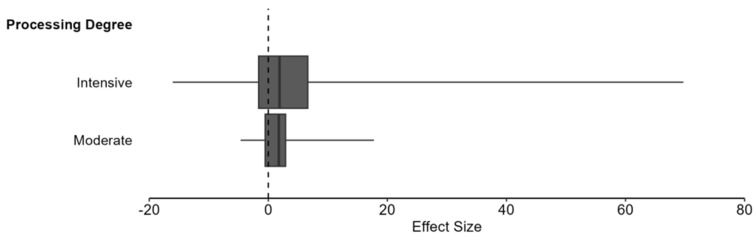
Forest plot of processing intensity on nutrient content. Effect size values beyond 0 represent a positive or negative effect based on the placement on the x-axis. A positive effect size represents a beneficial effect from processing degree, while negative effect sizes indicate a harmful effect from processing degree. The solid-black vertical lines are located at the mean for each processing degree and horizontal solid lines represent the 95% CI.

Beneficial effects account for 61.1% of the dataset, stemming from increased nutrient content, reduced LDL cholesterol, and reduced antinutrients. Harmful effects were present 14% of the time, resulting from a reduction in nutrients. Neutral effects occurred 12.1% of the time, showing no major impacts on nutrient content. The effect size across 137 study comparisons revealed an SMD = 1.405, 95% CI: 0.622 to 2.187, *p* < 0.001), indicating that processing provides a significant positive effect on nutrients (Figure 20). This translates to a modest improvement in nutrient content by 1.4 standard deviations when compared to unprocessed controls. The effects seen on nutrients were slightly smaller than processing impacts on digestibility (SMD = 1.971), which implies that processing has more impact on digestion than nutrition. However, the heterogeneity was extremely high (*I*^2^ = 95.76%, *τ*^2^ = 22.42, *Q*(136) = 920.24, *p* < 0.001), warranting a meta-regression to understand the true effects.

#### 6.4.3. Meta-Regression: Impact of Processing Intensity on Nutrient Contents

**Figure 21:**
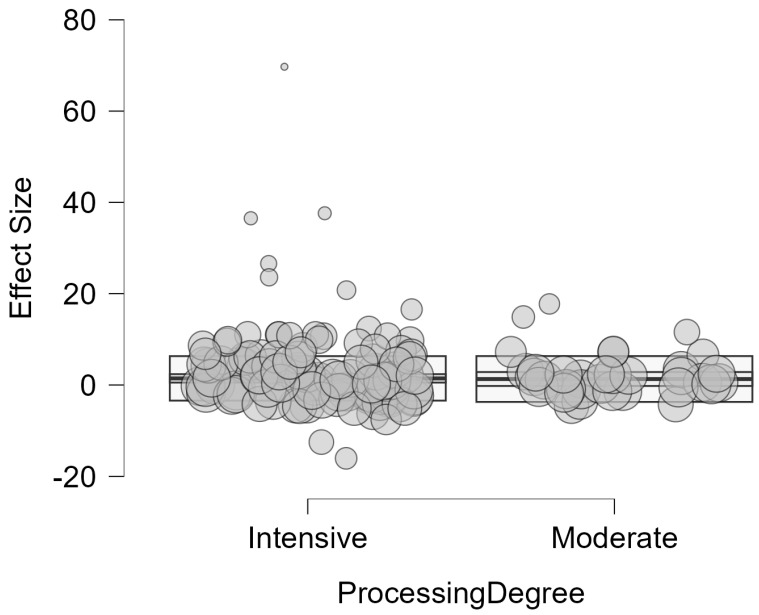
Bubble plots: impact of processing on nutrients. Each point represents the effect size from a single comparison, grouped by the degree of processing (intensive, minimal, moderate). The boxes show the median and interquartile range of effect sizes within each processing category, while whiskers indicate the range of values, summarizing the distribution of effects for each level of processing.

Moderation of nutrient retention by processing intensity showed no significant differences between groups, implying that moderate and intensive processing are relatively similar in outcomes (QM(1) = 0.34, *p* = 0.559). Intensive processing showed SMD = 1.548 (95% CI: 0.549 to 2.548, *p* = 0.003, k = 104), while moderate processing demonstrated SMD = 1.103 (95% CI: −0.058 to 2.265, *p* = 0.062, k = 33). These results suggest a potential for dose–response relationship as increases in intensity translated to increases in nutrient content. Although both categories trend toward positive effects, the moderate processing estimate includes zero in the confidence interval and does not reach statistical significance. Both groups maintained high heterogeneity and did not explain the observed variability between studies. The variability in effects can be seen in Figure 21, with clustering near effect sizes of zero.

#### 6.4.4. Meta-Regression: Impact of Ingredient Type on Nutrient Contents

Ingredient type emerged as the most significant moderator on nutrient content (QM(24) = 6644.92, *p* < 0.001), indicating that ingredient selection fundamentally determines the outcome of processing. Ingredients such as quinoa (SMD = 5.925, 95% CI: 3.080 to 8.771, *p* < 0.001), kiwicha (SMD = 5.635, 95% CI: 3.193 to 8.078, *p* < 0.001), barley (SMD = 2.974, 95% CI: 1.166 to 4.782, *p* = 0.002), and soybeans (SMD = 0.620, 95% CI: 0.254 to 0.986, *p* = 0.030) showed the largest improvements. These effects resulted from reductions to antinutrients and increases in antioxidant activity (Table 11). Negative impacts were also observed in ingredients such as sweet potatoes (SMD = −4.582, 95% CI: −5.320 to −3.845, *p* = 0.008), butternut squash (SMD = −3.561, *p* = 0.099), black beans (SMD = −0.789, 95% CI: −1.165 to −0.414, *p* < 0.001), and other legumes (chickpeas, lentils, navy beans, and yellow pea). The negative impacts were observed on triglycerides, antioxidant activity, and resistant starch content. Overall, processing impacts on nutrients are extremely ingredient-dependent.

#### 6.4.5. Meta-Regression: Impact of Processing Type on Nutrient Content

When moderating by processing method, statistical significance was reached (QM(18) = 199.02, *p* < 0.001), which reveals a true difference in effects when processing methods vary. Large positive effects were seen in methods such as germination (SMD = 8.679, 95% CI: 6.549 to 10.808, *p* < 0.001), steam explosion (SMD = 5.334, 95% CI: 0.695 to 9.974, *p* = 0.029), extrusion (SMD = 3.779, 95% CI: 1.106 to 6.453, *p* = 0.012), and extrusion + drying (SMD = 8.765, *p* = 0.119). Other methods produced null effects including cooking (SMD = 0.408, *p* = 0.820), extruded + fine milling (SMD = −0.071, 95% CI: −0.297, 0.156), and roasting (SMD = −0.659, *p* = 0.575). On the contrary, methods such as dehulling + boiling + milling (SMD = −1.223, 95% CI: −2.265, −0.181), blanching (SMD = −3.659, *p* = 0.089), and micronized fine milling (SMD = −1.501, 95% CI: −3.094, 0.094) produced negative effects on nutrient content. The impact of processing method on nutrient content is evident but pales in comparison to ingredient type.

#### 6.4.6. Heterogeneity and Variability: Impact of Processing Type on Nutrient Content

The analysis revealed high heterogeneity (*I*^2^ = 85.221%, *τ*^2^ = 5.732, *Q_e_*(136) = 920.24, *p* < 0.001), demonstrating a similar phenomenon as the digestibility assessment. This translates to 85% of observed variation stems from true between-study differences rather than sampling error. The substantial heterogeneity confirms that nutrient retention is highly context-dependent with variability outcomes dictated by ingredient type, nutrient type, and processing conditions. The H^2^ = 6.766 indicates that the observed variance is seven times greater than what would be expected from sampling error alone. Likewise, the wide 95% prediction interval is evident of substantial improvements or losses due to processing.

#### 6.4.7. Publication Bias Assessment: Impact of Processing on Nutrients

**Figure 22:**
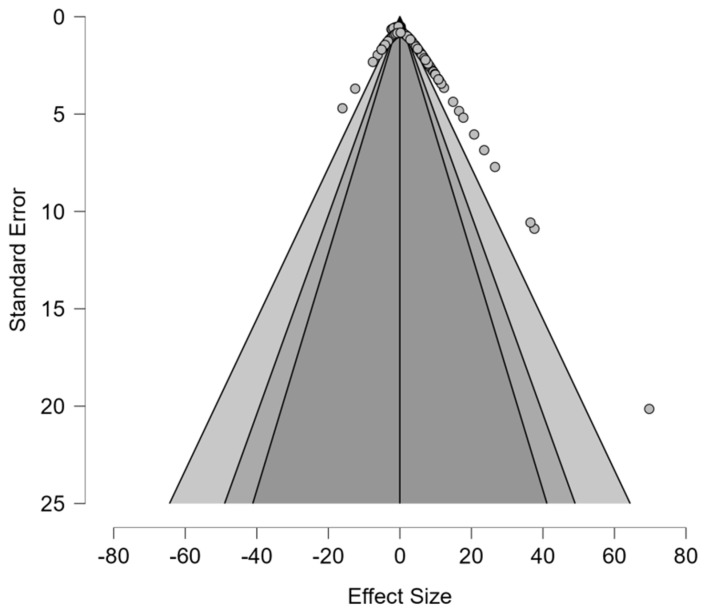
Funnel plot: impact of processing on nutrients.

Similarly to the assessment on digestion, the funnel plot asymmetry test in Table 12 revealed significant publication bias (*t*(137) = −2.329, *z* = −2.938, *p* < 0.001). This indicates the potential for smaller studies showing null or positive effects on nutrient content which are not represented by the published literature (Figure 22). However, the fail-safe N tests are evident of a robust effect size estimate, given that 137–5069 null studies would be needed to nullify the overall effect (Rosenthall fail-safe N = 5069, Orwin fail-safe N = 137, Rosenburg fail-safe N = 0).

### 6.5. Human-Grade vs. Feed-Grade Analysis

#### Dataset Overview and Scope

**Figure 23:**
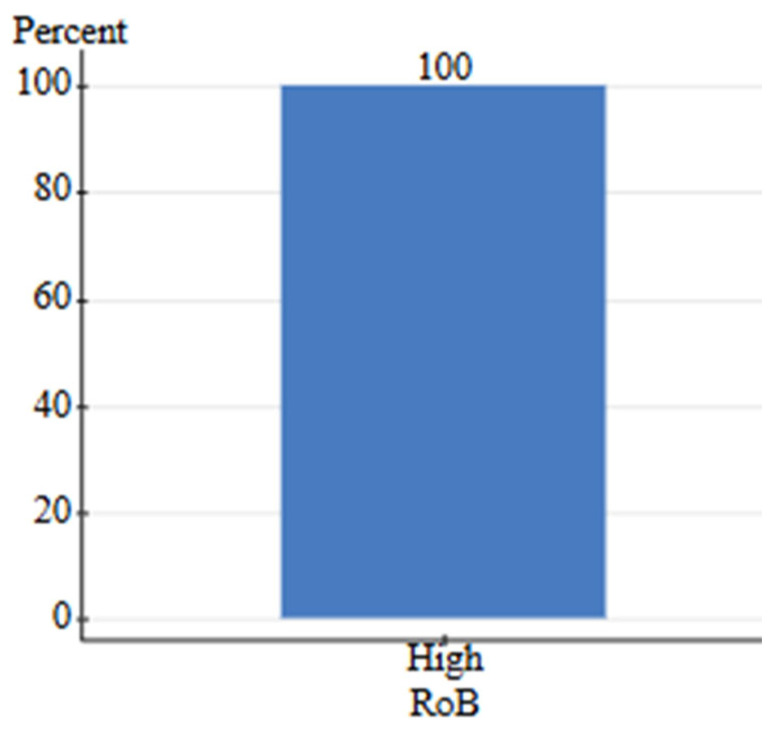
Relative frequency of bias: nutrition of human-grade ingredients.

**Figure 24:**
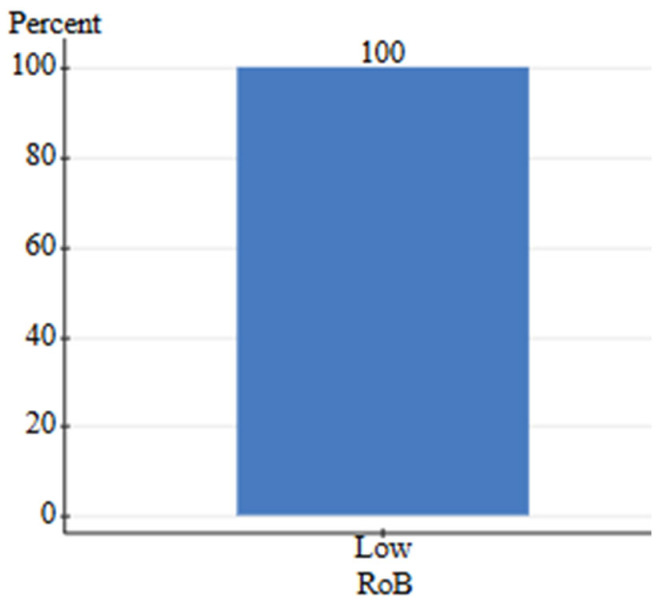
Relative frequency of bias: safety of human-grade ingredients.

The search criteria for human-grade and feed-grade foods provided six total studies, four of which were focused on nutritional differences, while two focused on safety. Nutritional studies comprised 75% B grade quality and 25% C grade quality studies. Safety studies contained one A grade study and one B grade study. Due to a high level of between-study heterogeneity and lack of identical outcomes measured, the studies were not assessed via meta-analysis. Instead, the pooled effect direction was calculated to understand the consensus of available studies. This was performed by assigning weight to each quality grade (3-A, 2-B, 1-C) and calculating a total score. The safety assessment for feed-grade vs. human-grade ingredients showed a 100% harmful effect direction (harmful score = 5, neutral score = 0, beneficial score = 0). For nutritional assessment, human-grade diets yielded an 85.7% beneficial effect direction, 14.3% neutral, and 0% harmful. On the contrary, results showed that 100% of nutrition-focused studies contained high detection of bias, while safety studies contained a 0% relative frequency for bias (Figure 23 and Figure 24). All nutritional studies showed signs of potential selective reporting bias and outcome measurement bias. This was due to industry funding, lack of blinding, and low randomization. Results should be interpreted carefully as scores do not fully reflect the impact of bias.

## 7. Discussion

### 7.1. Interpretation of Additive and Preservative Results

#### 7.1.1. Effects of Additives on Pet Adverse Events

According to the International Food Information Council, 62% of consumers report that ingredients have at least a moderate influence on their food purchasing decisions [184]. Likewise, 58% of consumers strongly or somewhat agree that they avoid products with “chemical sounding” ingredients [184]. The inclusion of additives in pet food is common throughout the pet food industry because of their ability to improve texture, appearance, palatability, and ensure adequate nutritive value. Some of the most common additives used in pet foods are flavor enhancers (e.g., turkey flavor or lactic acid), coloring agents (e.g., caramel color), and nutritional additives (e.g., vitamin D3), texture and binding agents (e.g., guar gum), probiotics and prebiotics (e.g., Inulin), emulsifiers (e.g., lecithin), and pH regulators (e.g., sodium bicarbonate). This study assesses the safety of additives in pet food by performing a risk difference Bayesian meta-analysis. Results show a pooled RD = 0.0006 (95% CI: −0.190, 0.892), supporting the null hypothesis of no effect on adverse events from additive consumption. High heterogeneity was observed, *I*^2^ = 73.31%, warranting subgroup analysis with moderators such as species, quality grade, duration length, additive type, and RoB. The BF for each inclusion ranged from BF = 0.066 to 0.552, indicating no moderator effects were detected. When moderating by additive type, effect sizes remained similar but standard error displayed clear differences between types. Functional additives appear to show potential for negative effects while nutritional additives show potential for positive effects. Zootechnical additives display an even distribution with potential for positive or negative effects. No evidence was found for the harms or benefits of additives being included in pet food diets.

#### 7.1.2. Effects of Preservatives on Pet Adverse Events

Pet food safety is the most important factor for 94% of consumers, making it critical for pet food manufacturers to provide preservatives that are regulated and proven safe [185,186]. Preservatives are used to prevent microbial activity in the form of humectants which control water activity to a level where microbes cannot utilize the available moisture to grow and cause spoilage. The other main type of preservative, antioxidants, are responsible for reducing oxidation that occurs in lipids and proteins exposed to oxygen which prevents harmful byproducts and nutrient degradation. The Bayesian RD meta-analysis assessed the current level of evidence for harm caused by preservatives, specifically any adverse event from vomiting to more extreme outcomes such as death. Results from this study are aligned with the null hypothesis that preservatives cause no meaningful harm to dogs or cats RD = 0.0003 (95% CI: −0.190, 0.892, BF = 0.162). A sensitivity analysis was performed to identify potential biases and the impact on effect size estimations, which showed no meaningful difference. Meta-regression by species, preservative type, duration, quality grade, and RoB provided no evidence for moderation. However, a trend emerged when moderating by species, as cats showed consistently higher effect sizes when compared to dogs, hinting at a potential species-specific biological mechanism (dogs RD = 0.229–0.339, cats RD = 0.434–0.601). Similarly, a trend emerged between preservative types as antimicrobials showed noticeably higher effect sizes and a wider range of uncertainty. This could represent a potential for harm specific to certain types of antimicrobials and warrants further research to understand the risk.

### 7.2. Interpretation of Human-Grade vs. Feed-Grade Results

The included studies [148,149,150,151] demonstrated highly relevant findings for this systematic review but revealed notable limitations that limit the comparability and interpretability of the findings. All four studies exhibited high levels of RoB due to an absence of blinding protocols across the outcome measurements, commercial funding from brands that used results to back claims, and utilization of non-canine models to compare with canine results. One particularly large issue was identified, as the human-grade formulas were not identical to feed-grade counterparts, which limits the direct comparability. For example, diets utilized in [150] showed a feed-grade Blue Buffalo recipe containing ingredients such as deboned chicken, chicken meal, brown rice, barley, oatmeal, pea starch, flaxseed, chicken fat, etc. However, the human-grade JustFoodForDogs formula contained chicken thighs, white rice, apples, carrots, spinach, chicken gizzard, chicken liver, etc. These non-identical recipe components yielded differing percentages of dry matter, protein, fat, fiber, nitrogen-free extract, and mineral content, which undoubtedly contributed to the differences in outcomes. As seen in Section 6.3.4, the ingredient differences account for the largest impact to digestion which limits the value of this comparison. Due to a small sample size, statistical significance was not reached by the majority of studies for serum chemistry, blood count, and fecal attributes [150]. Similar issues arose in a study by Algya et al. 2018, which concluded that fresh pet food was higher in protein and fat, and was well tolerated in the short term [187]. The authors found a noticeable microbiome shift, higher digestibility, and lower triglycerides [187]. These findings were confounded by a high risk of bias originating from industry funding, lack of blinding, selective reporting, and missing baseline data. Algya and colleagues stated that “differences due to the dietary treatments cannot be attributed to any specific ingredients or nutrient concentrations, but the diets as a whole”, which does not isolate variables and violates controlled experiment design principles [187]. Roberts and colleagues (2023) conducted an experiment to understand the nitrogen true metabolizable energy and amino acid digestibility differences between human-grade and feed-grade diets using precision-fed cecectomized rooster assays [148]. This study design provided high levels of control over confounding variables while advancing knowledge in vegan dog food digestibility. Issues arise when directly comparing the findings to amino acid digestion in dogs, as the measurement types are not identical. Studies [149,152] provide promising outcomes in dry matter digestibility of human-grade dog foods but lack variable isolation and blinding, which limit the significance of findings.

Aside from the lack of true comparison between feed-grade and human-grade foods, there is potential for human-grade ingredients to provide improved digestibility. Studies by Hendriks et al. (2013) and Oba et al. (2020) compare feed-grade digestibility values to diets made with human-grade ingredients [152,188]. Results show that average dry matter digestibility is identical between diets, while organic matter is slightly higher in human-grade diets (Table 13). While many values did not reach statistical significance, the human-grade diets trended towards higher protein digestibility aside from arginine (Table 13). However, this comparison is limited as the diets, animals, and measurements were different between studies. This lacks the ability to be used to draw true conclusions, and further research is needed to clarify the true effects.

A limitation of the search criteria was the lack of meat-related studies, which are a substantial aspect to pet diets. Faber et al. (2010) assessed the protein quality of meat and fish substrates in dog diets [189]. An immobilized digestive enzyme assay, cecectomized rooster assay, and ileally cannulated dog assay were utilized to assess the digestibility of each protein source. Results showed a significant difference in crude protein (CP) with pollock (96.9% CP) > salmon (92.8%) > chicken (90.3%) > pork (86.2%) > beef (82.7% CP). Pollock provided the highest digestibility across all assays while chicken breast remained the lowest (86% AA digestibility). The authors concluded that “despite the differences in composition and standardized digestibility values, when the protein sources were added to diets at a concentration of approximately 30%, no differences in test protein substrates were noted in either ileal or total tract nutrient digestibility [189]”. Findings from this study indicate that differences exist even between human-grade ingredients. A study by Kerr et al. (2012) studied digestibility, fecal characteristics, and metabolic response in cats that were fed a high-protein extruded diet, raw beef-based diet, and cooked beef-based diet [190]. Results from this study showed that no differences existed between the cooked and raw beef-based diets, while extruded diets had lower digestibility [190]. However, diets differed in composition which limits the true comparisons in metabolites, fecal scores, and digestion. While in vitro and standardized digestibility assays are able to predict differences in substrate composition and degree of processing, these differences frequently lack translation to clinically meaningful differences in pet digestibility when protein sources are incorporated into a complete and balanced diet.

The safety of human-grade ingredients could not be properly assessed with only two studies provided by the search criteria. A study by Spears and colleagues (2017) aimed to assess the chromium concentration of feed-grade ingredients such as corn, wheat, soybean meal, beet pulp, phosphates, and alfalfa hay [153]. The findings revealed chromium amounts that meet FDA 1.0 mg/kg limit compliance for 4/6 ingredients, while beet pulp amounts were at 122% of the FDA limit (1.222 mg/kg) and phosphates at 135% of the FDA limit (1.35 mg/kg) [153]. This represents a potential for risk in pets and warrants further studies to confirm it. A limitation of this study was the lack of human-grade comparisons which could have provided a perspective on the safety differences. The study by Tripathi et al. (2007) analyzed increasing feed-grade wheat concentrations and the association with Aflatoxin B1, a mycotoxin which is extremely harmful to pets [154]. This study included human-grade corn for comparison, containing 0 ppb of Aflatoxin B1. As wheat inclusion increased, the Aflatoxin B1 concentration increased linearly, with 100% wheat reaching 117.5% of the FDA limit (20 ppb/kg) [146]. While this result is indicative of harm, the 100% wheat recipe was not representative of inclusion levels found in typical pet food diets and should be interpreted with caution. The FDA establishes mycotoxin action levels for finished pet food (20 ppb/kg) with the Food Safety Modernization Act (FSMA), mandating preventative control to reduce this risk from occurring. The findings from this study should be confirmed by follow-up studies with more comparisons between human-grade and potentially commercial pet food diets to elucidate the true risk.

Feed-grade ingredients may contain a higher risk of having Biogenic Amines (BA), toxic byproducts of spoilage and bacterial contamination in meat products. A study by Montiegrove et al. (2023) found that higher inclusions of chicken meat meals resulted in linear increases to BA content, with large differences between fresh meat and meat meals [191]. This represents a potential risk in commercial pet foods which commonly utilize meat meals. Pinto et al. (2023) found similar results when studying hydrolyzed chicken liver and chicken byproduct meal, with the chicken meal diet containing 11.5× the BA content compared to the hydrolyzed liver diet [192]. These studies pinpoint an underlying risk to feed-grade meat meals that warrants further examination.

### 7.3. Interpretation of Whole vs. Processed Ingredient Results

#### 7.3.1. Effects of Processing on Digestibility

The impact of processing on digestibility is far overshadowed by the focus on nutritional content. Digestion is critical for pet health as undigested nutrients cannot be utilized by the body, creating deficiencies and contributing to negative health outcomes [193,194]. This study aimed to uncover those impacts while establishing a general effect direction. The random-effects meta-analysis found statistical significance in the positive impacts of processing on digestion (SMD = 1.971, 95% CI: 0.567, 3.374, *p* = 0.005). The largest contributors to the benefits of processing included ingredient type and processing method, both explaining the substantial heterogeneity between studies. The type of ingredient selected was the most crucial factor in determining digestibility outcomes as the effect sizes ranged from −4.477 to 8.419, meaning future studies may see large benefits or harms to digestibility. Similarly, the processing method of choice was responsible for large impacts on digestibility outcomes. The differences between methods are critical knowledge gaps that will need to be answered prior to substantiating claims around processing benefit or harm. Contrary to typical beliefs, these findings suggest that processing intensity is trivial when predicting digestibility outcomes (*p* = 0.562). As with nutrient content, formulators should prioritize method type and ingredient selection rather than how aggressively processing is applied. Future research can address this topic directly by measuring digestibility across multiple temperatures, durations, and pressures using the same ingredients and ratios of those ingredients.

#### 7.3.2. Effects of Processing on Nutrient Content

As pets are viewed as members of the household, owners expect their food to be nutritious and support a long, healthy life. However, much of the nutritional aspects of pet food are not clear to consumers aside from claims made by companies they trust. Processing emerges as one of the aspects that consumers perceive as unhealthy or contributing to disease [185]. The results from this study do not prove this phenomenon to be true, with a variety of effects and levels of severity which are present when processing pet food ingredients. This study found that a statistically significant positive association exists within the 137 included comparisons (SMD = 1.405, 95% CI: 0.622–2.187, *p* < 0.001). This finding is accompanied by high levels of heterogeneity (*I*^2^ = 85.221%, Q(136) = 920.24, *p* < 0.001, *τ*^2^ = 5.732), necessitating subgroup analysis by moderators to understand the true effects of processing. One of the most meaningful findings included the impact of ingredient type on the processing effect direction (QM(24) = 6644.92, *p* < 0.001). This result indicates that processing is almost entirely dependent on the outcome being measured (protein quality, antioxidant activity, and fat content) and composition of the ingredients (macronutrients, micronutrients, and antinutrients). Processing method was found to be another significant factor in nutrient retention (QM(18) = 199.02, *p* < 0.001). Many studies assess the impact of processing intensity on the nutritional content of a diet but overlook the magnitude of the effect of the methods themselves [15,195,196]. Interestingly, the processing intensity was not a significant moderator of nutrient content (QM(1) = 0.34, *p* = 0.559). The processing method and ingredient type are vital factors in understanding whether effects are beneficial or harmful to pet food. Future research in this area should utilize identical processing conditions across multiple differing ingredients to understand their true differences. Likewise, testing should be conducted on identical finished products across multiple methods of processing to understand impacts on nutrient content. This evidence does not provide a definitive answer to the debate, instead serving as an indicator that more high-quality studies are needed.

### 7.4. Justification of Methodological Approach

Datasets for each of the analyses were plagued with high levels of heterogeneity due to study design differences, outcome measures, population, and intervention differences that could not be controlled. Likewise, controlling via sub-analyses presented another issue in which important studies would be excluded, changing the effect direction and not representing the overall effect in a comprehensive manner. This would have limited the ability to broadly answer each of the research questions posed.

In order to obtain an accurate representation of the true harm caused by additives and preservatives, a Bayesian risk difference meta-analysis with meta-regressions was selected to accurately display effect sizes without inflating results. This method demonstrated consistency with the rate of adverse events between treatment and control groups. The Peto meta-analytic method, paired with the Mantel–Haenszel weighting, provided a model-free assessment of observed heterogeneity. The high *I*^2^ was indicative of true heterogeneity between studies, motivating investigation of moderating factors and necessitating random-effects modeling rather than relying on the pooled estimate alone.

Random-effects meta-analysis using Standard Mean Difference was selected to assess the impact of processing on nutrient retention and digestion. This methodology was able to handle the inherent heterogeneity expected across diverse ingredient types, processing methods (extrusion, wet roasting, germination, and thermal treatment), processing intensities, analytical procedures, and study designs. These random-effects models are preferred when studies contain different underlying effects that form a distribution around the pooled effect, which is more accurate than assuming one single effect estimate to represent the dataset. Since the majority of studies reported mean outcomes per ingredient and processing method, SMD was utilized to align all studies under a singular measure, allowing us to classify changes as beneficial, neutral, or harmful.

Similarly to the additive/preservative and processing studies, human-grade food assessments were scarce and extremely heterogeneous. However, the RoB presented more challenges in this dataset which could not be included in a meta-analysis as the results would be misleading and likely inaccurate. Further studies are needed to create a proper assessment. Instead, the discussion (Section 7.2) provided study-level insights that were not demonstrated via statistical methods.

Typical systematic reviews and meta-analyses are accompanied by a GRADE assessment, which establishes a quality of evidence using consistent methodologies. The authors chose to use aspects of the Cochrane GRADE assessment to assign a score to each study, allowing for exclusion prior to meta-analysis. This method better suited the high heterogeneity between studies and generalized research questions, while maintaining a rigorous analysis.

### 7.5. Limitations

The selected approaches were able to generate answers to each research question and deliver insights not previously covered in the available literature. However, there were many limitations that should be recognized prior to interpreting these findings as definitive.

The heterogeneity observed across studies was substantial, particularly in the processing analyses where *I*^2^ values exceeded 87% for both digestibility and nutrient content outcomes. While meta-regression identified ingredient type and processing method as significant moderators, considerable unexplained variance remained. This heterogeneity stemmed from differences in study designs, outcome measures, analytical methods, ingredient matrices, processing parameters, and population characteristics. The diversity of these factors limited the ability to isolate specific causal mechanisms and reduced the precision of pooled effect estimates.

The human-grade versus feed-grade ingredient analysis suffered from critical methodological constraints. Only six studies met inclusion criteria, and all nutritional comparison studies exhibited high risk of bias due to industry funding, lack of blinding protocols, and selective outcome reporting. More importantly, the compared diets were not formulated with identical ingredient profiles, nutrient compositions, or processing methods, confounding the attribution of observed differences to ingredient grade alone. The lack of standardized definitions for human-grade and feed-grade ingredients across studies further complicated comparisons and limited the generalizability of findings.

Publication bias was detected in the processing analyses, with funnel plot asymmetry tests indicating that smaller studies with null or negative findings may be underrepresented in the published literature. While fail-safe N analyses suggested robustness of the overall effect estimates, the systematic underreporting of non-significant results could inflate the perceived benefits of processing on digestibility and nutrient content. Similarly, conflicting results between asymmetry tests in the preservative analysis suggest potential selective publication patterns that require additional investigation.

The scope of this review was limited to English-language publications available in three major databases, potentially excluding relevant research published in other languages or less accessible journals. Similarly, the restriction to full-text articles may have omitted pertinent conference proceedings, gray literature, or ongoing studies that could inform the current understanding of fresh pet food claims.

The processing analyses focused mainly on individual ingredients rather than complete diets, limiting the direct applicability of findings to finished pet food products. The interactive effects of multiple ingredients, processing sequences, and formulation parameters in commercial pet foods may produce outcomes that differ substantially from those observed in this single-ingredient studies. Additionally, the classification of processing intensity into minimal, moderate, and intensive categories, while systematic, involved subjective judgments about threshold values and could not capture the full complexity of industrial processing protocols. Likewise, minimal processing was extremely underrepresented as a majority of studies did not include minimally processed variants for comparison.

These limitations collectively indicate that while this systematic review provides the most comprehensive synthesis of evidence currently available regarding fresh pet food claims, definitive conclusions remain constrained by the quality, quantity, and design of the underlying research base. Future investigations employing rigorous experimental designs, standardized outcome measures, adequate sample sizes, long-term follow-up periods, and transparent reporting practices are essential to substantiate or refute the marketing claims evaluated in this review.

## 8. Conclusions and Recommendations

### 8.1. Conclusions

Large improvements have been made to pet food to optimize the health of pets since the first commercial diets were produced in 1922 and 1956 [197]. The purpose of this systematic review was to establish a scientific consensus of fresh pet food claims and clearly identify research gaps for future studies to explore.

The analysis of 60 additive studies and 39 preservative studies using Bayesian meta-analysis revealed pooled risk differences of 0.0006 and 0.0003, respectively, with Bayes factors providing strong evidence for null hypotheses. The findings do not support claims that approved additives and preservatives cause harm when used within regulation-compliant levels. The adverse event rates between treatment and control groups were nearly identical, attributing the differences to normal biological variations rather than toxicity from ingredients. Meta-regressions found no significant moderation by species, study duration, quality grade, or risk of bias, further supporting the lack of true effects.

The analysis of the effect of processing on ingredients encompassed 102 digestibility comparisons and 137 nutrient comparisons, demonstrating significant pooled effects (SMD = 1.971 and SMD = 1.405, respectively). These estimates were plagued by extreme heterogeneity as ingredient type and processing method were found to be large contributors to the large effect size ranges. Ingredient type emerged as the most significant moderator of processing outcomes, with variations causing large fluctuations in outcomes. Likewise, processing methods were significant moderators of both digestion and nutrient content. Processing intensity showed no significant moderation of outcomes. These findings indicate that processing effects are highly context-dependent and cannot be generalized across all ingredients or pet food formulations.

The human-grade versus feed-grade ingredient comparison yielded only six eligible studies, all of which exhibited methodological limitations that prevented definitive conclusions. The nutritional comparison studies showed high risk of bias due to industry funding, absence of blinding protocols, and non-identical formulation parameters between compared diets. The confounding of ingredient grade with differences in nutrient profiles, ingredient compositions, and processing methods made it impossible to isolate the specific effects of ingredient grade on pet health outcomes. While safety assessments suggested that feed-grade ingredients may contain higher contaminant levels, the limited sample size and lack of standardized definitions for human-grade and feed-grade ingredients across studies constrained the generalizability of these findings.

Overall, current scientific evidence does not substantiate the widespread marketing claims that additives and preservatives are harmful to companion animals when used within regulatory guidelines. In the context of whole and processed ingredients, the evidence found in this study does not support this claim and varies substantially by ingredient type and processing method. The superiority of human-grade ingredients over feed-grade ingredients remains inadequately investigated, with existing studies confounded by multiple variables that prevent causal attribution.

### 8.2. Recommendations

#### 8.2.1. Industry Professionals and Pet Food Manufacturers

Pet food companies should ensure that all marketing claims are backed with accessible and peer-reviewed scientific evidence before conveying them to consumers, allowing for informed decisions to be made. Minimally processed diets should be assessed against identical ingredient matrices or similar formulations to draw more conclusive evidence. Likewise, these studies necessitate larger sample sizes to sufficiently power the analytical tests to statistical significance. Comprehensive prospective studies are needed to establish whether processing intensity causally affects long-term health outcomes in companion animals across their lifespan, employing standardized outcome measures and controlling for ingredient composition, nutritional intake, and variability between pets.

#### 8.2.2. Regulatory Agencies

The FDA and FTC should establish clear guidelines for the use of marketing terms such as “fresh,” “human-grade,” “whole ingredients,” and “minimally processed” to prevent misleading claims that lack standardized definitions. Enforcement actions should target companies making unsubstantiated health claims or employing fear-based marketing strategies that disparage safe and approved ingredients without scientific justification.

#### 8.2.3. Veterinary Professionals

Veterinarians should base dietary recommendations on peer-reviewed scientific evidence rather than paid sponsorships or marketing claims. This improves consumer trust in recommendations made by veterinary professionals while improving the lives of pets due to optimization of diet. Dietary counseling should emphasize the quality of ingredients and processing methods rather than the degree of processing that took place during manufacturing. Furthermore, veterinary professionals should inform consumers of risks to feeding homecooked diets and the need for complete and balanced nutrient profiles which are regulated by regulatory agencies.

## Figures and Tables

**Table 1:** Assessment of additive studies.

Study ID: [Unique Identifier]	Species: [Dog/Cat/Mixed]	Additive Type: [Specific Compounds]	Exposure Level: [Dose/Concentration]	Outcomes: [Primary/Secondary Endpoints]	Effect Direction: [Harmful/Beneficial/Neutral]	Key Findings: [Brief Summary]	Limitations: [Major Concerns]	Risk of Bias [Low/High]	Quality Grade [A, B, C, D, F]
Soares et al., 2023 [43]	Dog	Yeast cell wall and oregano essential oil	1.5–3.0 kg/ton	Digestibility, Fecal ammonia/pH	Neutral	No adverse reactions up to 3 kg/ton; only dry matter digestibility and fecal ammonia significantly changed	Only two endpoints with short feeding	Low	B
Panja et al., 2023 [44]	Dog	Lactobacillus probiotic (multi-strain)	10^9^ CFU/dog	Fecal score, creatinine	Neutral	One strain increased creatinine, otherwise neutral	Few inflammatory markers studied	Low	B
El-Wahab et al., 2023 [45]	Dog	Powdered/granulated cellulose, lignocellulose	2 g/kg BW	Digestibility, fecal score	Neutral	Wet fecal output is higher with cellulose, energy digestibility lower	Short period; few endpoints	Low	B
Cabrita et al., 2023 [46]	Dog	Microalgae (Chlorella, Nannochloropsis, Tetradesmus)	0.5, 1.0, 1.5% of diet	Fecal metabolites, VFA, pH, score	Beneficial	No adverse effects at all tested levels	Short feeding	Low	B
Jackson et al., 2023 [47]	Dog	Medium-chain triglycerides (MCT), fish oil, and combinations	Various concentrations in diet formulations	Serum metabolites, blood chemistry panels, palatability	Neutral	No adverse changes in blood chemistry parameters; good palatability of supplemented diets	Relatively short study duration; limited to healthy adult dogs	Low	B
Nascimento et al., 2022 [48]	Dog	Herbal choline source (polyherbal blend)	765 mg/kg diet	Blood biochemistry, cardiac function parameters, gene expression	Beneficial	No adverse effects; potential cardiovascular and metabolic benefits based on gene expression profiles	Mechanistic study with limited clinical endpoint assessment	Low	B
Berk et al., 2022 [49]	Dog	Medium-chain triglycerides (MCT oil)	5.5% of dry matter intake	Gastrointestinal tolerance, food intake behavior, palatability	Neutral	No gastrointestinal adverse effects; increased food intake time but no preference differences	Short study duration; limited to healthy dogs	Low	B
Mendoza-Martinez et al., 2022 [50]	Dog	Polyherbal choline source (phosphatidylcholine)	200, 400, and 800 mg/kg diet	Blood metabolites, gene expression analysis, body weight changes	Beneficial	Gene expression changes indicated benefits for cardiovascular/metabolic health, cancer prevention, immune function	Transcriptomic endpoint focus; limited traditional clinical measures	Low	A
Mendoza-Martinez et al., 2021 [51]	Dog	Polyherbal phosphatidylcholine blend	200–800 mg/kg diet	Apparent digestibility coefficients, fecal consistency scores	Neutral	No significant differences in digestibility or fecal scores between treatment and control groups	Limited outcome measures; focus only on digestive parameters	Low	B
Park et al., 2019 [52]	Dog	Fermented medicinal plant extracts	1% of total daily food intake	Fecal bacterial populations, food preference, fecal quality	Beneficial	No adverse effects; improved beneficial bacterial populations in feces	Short study duration; limited clinical relevance of microbiome changes	Low	B
Yang Sun et al., 2019 [53]	Dog	Weissella cibaria probiotic strain	50 g probiotic preparation daily	Apparent digestibility, fecal microbiology, immune parameters	Beneficial	No adverse effects; improved digestibility and beneficial fecal microflora changes	Short feeding trial; limited to healthy dogs	Low	B
Santos et al., 2017 [54]	Dog	Papain enzyme supplement	2.28 million units of papain enzyme activity	Apparent digestibility coefficients, fecal characteristics	Beneficial	No adverse effects; improved protein digestibility with papain supplementation	Short study duration; limited to digestibility parameters	Low	B
Santos et al., 2013 [55]	Dog	Yucca schidigera extract and zeolite	125–350 ppm yucca extract + 0.5–1.0% zeolite	Apparent digestibility, fecal ammonia, mineral excretion	Neutral	Potential concern for increased calcium excretion at higher zeolite levels; reduced fecal ammonia.	Short study duration; mineral balance concerns at higher inclusion rates; did not reach statistical significance	Low	B
Aquilina et al., 2013 [56]	Dog	Cylactin (Enterococcus faecium preparation)	4.5 × 10^6^ to 2.0 × 10^9^ CFU/kg feed	Fecal consistency scores, immune parameters (IgA levels)	Neutral	Inconsistent fecal score improvements; potential beneficial effects on intestinal IgA concentrations	Variable study protocols; inconsistent results across trials	Low	B
Aquilina et al., 2013 [56]	Cat	Cylactin (Enterococcus faecium preparation)	4.5 × 10^6^ to 2.0 × 10^9^ CFU/kg feed	Fecal consistency scores, immune parameters (IgA levels)	Neutral	Inconsistent fecal score improvements; potential beneficial effects on intestinal IgA concentrations	Variable study protocols; inconsistent results across trials	Low	B
Stoeckel et al., 2011 [57]	Dog	Fish oil (DHA-rich supplement)	85 mg/kg body weight daily	Erythrocyte membrane fatty acid composition, incorporation kinetics	Beneficial	No adverse effects; effective incorporation of n-3 fatty acids into cell membranes	Limited to healthy dogs; focus on fatty acid incorporation rather than clinical outcomes	Low	B
Lovern et al., 2001 [58]	Dog	Low sodium diet + Furosemide (diuretic pharmaceutical) + NaCl (1% Na and 2.2% Cl)	2 mg/kg body weight	Plasma drug concentrations, pharmacokinetic parameters	Beneficial	No adverse effects at therapeutic dose; established pharmacokinetic profile	Small control sample size; 1 dog dropped out due to refusal to eat diet	Low	C
Wang et al., 2024 [59]	Cat	Bifidobacterium longum probiotic	0.16% of diet (10^8^ CFU/g)	Blood biochemistry, fecal microbiome analysis, immune markers	Beneficial	No adverse effects; improved beneficial gut bacteria populations and immune function markers	Short study duration; limited to healthy cats	Low	B
Gonzalez et al., 2023 [60]	Cat	Yeast cell wall compounds	0.15–0.3% of diet	Fecal microbiota and metabolites	Neutral	None at 0.15–0.3% of diet	Short study period but randomized block design was effective	Low	A
De Oliveria Matheus et al., 2021 [61]	Cat	Saccharomyces cerevisiae	0.3–0.6% of diet	Fecal microbiota and immunology	Neutral	None at 0.3–0.6%	Single-sex study; 3 cats did not consume enough food and were excluded	Low	A
King et al., 2014 [62]	Cat	Lanthanum carbonate phosphate binder (Lenziaren)	0.5–1.0 g per day	Urine phosphorus concentrations, serum phosphorus, palatability	Beneficial	No adverse effects; effective phosphorus binding with dose-dependent response	minor clinical signs which were judged to be unrelated to the test item, e.g., conjunctivitis; loose feces or otitis externa, all cats remained in good health throughout the study	Low	B
Pires et al., 2013 [63]	Cat	Urinary acidifiers (ammonium chloride, DL-methionine)	0.3%, 0.6%, and 0.9% of dry matter	Urine pH, apparent digestibility, blood chemistry, mineral balance	Beneficial	No adverse effects up to 0.9% inclusion; effective urine acidification without metabolic disruption	Healthy cats only; short treatment periods. Hyponatremia and hypochloremia but these were attributed to diet composition as these were present across all groups	Low	B
Schmidt et al., 2012 [64]	Cat	Lantharenol phosphate binder (aluminum hydroxide-based)	Up to 16 g/kg body weight daily	Apparent digestibility, serum phosphorus levels, palatability	Beneficial	Well tolerated and safe up to maximum dose; effective phosphorus binding without affecting other nutrients	Short study duration; healthy cats only; limited clinical endpoint assessment; repeated vomiting in 7 cats at highest dose in study 1	Low	A
Kelley et al., 2009 [65]	Dog	Bifidobacterium animalis AHC7 probiotic	Up to 2 × 10^10^ CFU daily	Apparent digestibility, fecal consistency scores, immune parameters	Beneficial	No adverse effects at any dose level; improved fecal quality and potential immune benefits	Healthy dogs only; relatively short study duration	Low	B
Szweda et al., 2014 [66]	Dog	lansoprazole, liquorice extract, and a herbal solution	50 mg/kg body weight daily	Gastrointestinal histopathology, clinical improvement, adverse effects	Beneficial	No adverse effects; improved gastrointestinal histopathology and clinical signs	Small treatment groups and less focus on liquorice extract/herbal solution; “Occasional diarrhea and blood in the feces” were observed, but these were noted across “dogs from any of the groups” (not specifically attributed to individual groups)	Low	C
Barrouin-Melo et al., 2016 [67]	Dog	Fish oil vs. corn oil supplementation	0.2 mL/kg body weight per day	Oxidative stress markers, serum biochemistry, hematology, clinical signs	Beneficial	Fish oil group showed improved antioxidant status and reduced oxidative stress compared to corn oil	Small sample size; no placebo control group; limited clinical outcome measures. One dog developed hemorrhagic enteritis and was excluded; one dog was euthanized at the owner’s request due to deteriorating clinical condition	High	B
Martí-Angulo et al., 2014 [68]	Dog	Hyaluronic acid and collagen supplement	1 tablet daily (200 mg chondroitin + 312.5 mg glucosamine)	Radiographic assessment of elbow dysplasia, clinical evaluation	Beneficial	Reduced incidence of elbow dysplasia and improved clinical signs in supplemented group	Breed-specific study; long study duration required; subjective clinical assessments	Low	B
Nganvongpanit et al., 2009 [69]	Dog	Doxycycline (tetracycline antibiotic) vs. Chondroitin sulfate	4 mg/kg/day orally (2 mg/kg twice daily)	Clinical scores (lameness, joint mobility, pain, weight-bearing), radiographic changes, serum biomarkers (CS-WF6 epitope, hyaluronan)	Beneficial	Significant improvements in lameness, joint mobility, pain, and weight-bearing (*p* < 0.05) at multiple time points; biomarker improvements at 2–3 months	Breed-specific study (Golden/Labrador retrievers); comparison to chondroitin sulfate rather than placebo	Low	B
Pena et al., 2014 [70]	Dog	Mitratapide (microsomal triglyceride transfer protein inhibitor) vs. low-fat high-fiber diet	0.63 mg/kg daily for 2 × 21 days with 14-day washout	Body weight, blood pressure, metabolic parameters (cholesterol, triglycerides, glucose, liver enzymes)	Beneficial	Significant reductions in diastolic BP (*p* < 0.001), total cholesterol (*p* = 0.027), and ALT (*p* = 0.014) vs. diet alone; similar weight loss in both groups	No placebo control; limited to obese dogs only	High	B
Takenaka et al., 2017 [71]	Cat	Beraprost sodium (prostacyclin analog)	55 μg/cat twice daily (110 μg/day total)	Serum creatinine, serum phosphorus-to-calcium ratio, urine specific gravity, clinical signs	Beneficial	BPS inhibited serum creatinine increase vs. placebo (*p* = 0.0071); well tolerated with minimal adverse effects	Single adverse event (vomiting) in placebo group; limited to cats with stable CKD	Low	A
Rose et al., 2017 [72]	Dog	Synbiotic supplement (probiotic + prebiotic combination)	Commercial synbiotic preparation (specific dose not detailed)	Diarrhea incidence, shelter welfare metrics, cost analysis	Beneficial	Significant reduction in diarrhea incidence; improved welfare outcomes; cost-effective intervention	Variable shelter stay duration; heterogeneous population;	Low	A
Bybee et al., 2011 [73]	Dog	Enterococcus faecium SF68 probiotic	1 g daily (2.1 × 10^9^ CFU/g)	Fecal score and diarrhea presence	Beneficial	Cats fed SF68 had fewer episodes of diarrhea ≥2 days (7.4%) compared to placebo (20.7%); *p* = 0.0297	Statistical differences between dogs were not detected; dynamic shelter population; 28 dogs had at least 1 episode of diarrhea	Low	A
Bybee et al., 2011 [73]	Cat	Enterococcus faecium SF68 probiotic	1 g daily (2.1 × 10^9^ CFU/g)	Fecal score and diarrhea presence	Beneficial	Puppies fed SF68 were compared with a placebo group and were shown to have statistically greater total plasma IgA concentrations (*p* < 0.05), numerically greater fecal IgA concentrations (*p* = 0.056), and increased canine distemper virus-specific plasma IgG and IgA concentrations over time after vaccination	Statistical differences between dogs were not detected; dynamic shelter population; 34 cats had at least 1 episode of diarrhea	Low	A
Lascelles et al., 2010 [74]	Cat	EPA/DHA omega-3 fatty acids, green-lipped mussel extract, glucosamine/chondroitin sulfate	High EPA/DHA content diet + supplements	Activity monitoring (accelerometry), subjective owner/vet assessments, blood EPA/DHA levels	Beneficial	Significant increase in activity in test-diet group (*p* < 0.001); control group activity declined significantly (*p* < 0.001)	Subjective assessment methods need validation; relatively short study period	Low	B
Center et al., 2000 [75]	Cat	L-carnitine	250 mg/cat/day orally	Weight loss, amino acids, carnitine, safety	Beneficial	Faster fat loss, safe, increased plasma markers	No body composition (fat/lean) measures	Low	A
Freeman et al., 1998 [76]	Dog	Fish oil	Exact dosage not specified	Body composition, cytokine concentrations, fatty acids, survival	Beneficial	Fish oil supplementation decreased IL-1 concentrations and improved cachexia; IL-1 reductions correlated with survival	Relatively small sample size	Low	A
Theisen et al., 1997 [77]	Cat	Potassium gluconate	4 mEq/d	Muscle potassium content, glomerular filtration rate (GFR), effective renal plasma flow (ERPF)	Beneficial	Cats with CRF had significantly lower muscle potassium content than controls before treatment	Small sample size, no significant differences between treatment groups	Low	A
Singh et al., 2015 [78]	Cat	Acarbose	25 mg/cat daily	Postprandial glucose	Beneficial	Improves glycemic control with high-carb meals	Short-term, healthy cats, not diabetic	Low	A
Freiche et al., 2011 [79]	Cat	Psyllium (fiber)	Enriched dry diet (11–11.5% fiber)	Fecal consistency, appetite, body weight	Beneficial	Diet effective for constipation, well tolerated	No RCT; open-label; one instance of vomiting in trial 1	High	C
Tam et al., 2011 [80]	Cat	Polyethylene glycol 3350 (PEG3350)	1.9 g per meal, twice daily (titrated to effect)	Fecal scores, safety parameters, palatability	Beneficial	PEG3350 was safe and effective as an oral laxative in cats	Very small sample size; three cats developed mild hyperkalemia (5.6–6.0 mmol/L, RI 3.9–5.5 mmol/L)	Low	C
Rishniw and Wynn 2011 [81]	Cat	Synbiotic (Azodyl) vs. prebiotic psyllium husk	As per label (sprinkled, not caps)	BUN, creatinine	Neutral	Sprinkled product had no CKD benefit	Narrow scope and low sample size; 1 cat (euthanized due to worsening renal failure)	Low	C
Hall et al., 2008 [82]	Cat	Low-carb high-protein (LCHP) veterinary diet vs. control	Assigned diet, both canned/dry	Glycemic control, remission	Beneficial	LCHP diet + glargine led to lower fructosamine; remission in 2/12; good control	Small n, over-the-counter control diet not truly high-carb, short duration. One cat died of hepatic failure and diabetic ketoacidosis	Low	B
Gunew et al., 2008 [83]	Cat	Meloxicam vs. control	0.01–0.03 mg/kg PO once daily	Safety assessment, efficacy evaluation, palatability, creatinine	Beneficial	Long-term meloxicam safe and effective for feline osteoarthritis with minimal adverse effects	Open-label design; subjective owner assessments; 2 cats experienced gastrointestinal upset (vomiting)	Low	B
Maggs et al., 2007 [84]	Cat	Lysine	11 or 51 g/kg diet	URD signs, viral DNA, plasma AA	Neutral	No difference in disease scores overall	Basal vs. high lysine; narrow scope	Low	B
Appleton et al., 2002 [85]	Cat	Chromium tripicolinate	0, 150, 300, 600 ppb	Glucose/insulin tolerance	Beneficial	Dose-dependent improvement in tolerance; no insulin changes	Small sample/group; short trial	Low	A
Nelson et al., 2000 [86]	Cat	Dietary insoluble fiber (powdered cellulose)	12% cellulose dry-matter, crossover	Glycemic control: glucose, HbA1c, weight	Beneficial	High fiber significantly reduced glucose and HbA1c, neutral on weight and insulin	Palatability, mild constipation; 3 cats died or were euthanized (lymphoma, chronic pancreatitis, trauma) and 3 cats were removed (2 acromegaly, 1 renal failure) during the study; none of the 6 adverse events in the control group were treatment-related	Low	A
White et al., 2017 [87]	Dog	Multi-strain probiotic + Standard therapy	20 mL/kg daily	Mucosal microbiota, clinical remission, tight junction protein expression	Neutral	Probiotic therapy was associated with upregulated expression of tight junction proteins suggesting beneficial effects on mucosal homeostasis	Similar microbiota effects to standard therapy alone; 5 dogs were withdrawn (1 euthanized for refractory protein-losing enteropathy, 3 diagnosed with intestinal neoplasia, 1 lost to follow-up) but none were attributable to the study therapy	Low	A
Templeman et al., 2018 [88]	Dog	Tryptophan	0.05%, 0.1%, and 0.15% supplementation on top of 0.18% basal level	Behavioral parameters (activity, distance, confidence, ear position)	Neutral	Graded tryptophan supplementation did not significantly affect behavior in response to familiar/unfamiliar individuals	Health parameters were not measured but no adverse reactions were noted	Low	B
Freeman et al., 1997 [89]	Cat	Magnesium chloride	Up to 9.2 mg/kg BW	Serum/urine creatinine and magnesium	Neutral	None up to 9.2 mg/kg BW	Three interrelated studies with narrow scope	Low	B
Segarra et al., 2016 [90]	Dog	Chondroitin sulfate and prebiotics (resistant starch, β-glucans and mannaoligosaccharides)	Dosage not specified	Canine IBD activity index (CIBDAI), histologic score, serum biomarkers	Beneficial	Combined supplement with hydrolyzed diet was safe and induced improvements in selected serum biomarkers	Study was likely underpowered	Low	A
Hielm-Björkman et al., 2012 [91]	Dog	Deep sea fish oil (EPA/DHA)	1 mL/5 kg BW = ~90 mg/kg EPA	Pain, mobility, NSAID use, QOL	Neutral	No stat. benefit vs. placebo, but improved VAS, pain, QOL within fish oil group	Heterogeneous OA; partial blinding (oil flavor/smell); narrow scope; one dog experienced a serious adverse event (hemorrhagic enteritis) and was withdrawn from the study	High	B
Marshall-Jones et al., 2006 [92]	Cat	Lactobacillus acidophilus DSM13241	2 × 10^8^ CFU/d	Fecal bacterial populations, hematologic analysis, immune function	Beneficial	Probiotic altered gastrointestinal microflora balance and resulted in beneficial systemic and immunomodulatory effects	Study in healthy cats only; crossover design	Low	A
Stiles et al., 2002 [93]	Cat	L-lysine monohydrochloride	500 mg twice daily	Clinical scores for conjunctivitis, virus isolation, plasma lysine, and arginine concentrations	Beneficial	Oral L-lysine resulted in less severe manifestations of conjunctivitis caused by FHV-1	Small sample size and short study duration	Low	C
Funaba et al., 2003 [94]	Cat	Ammonium chloride	69 mmol/kg diet	Struvite activity product and urine pH	Beneficial	None at 69 mmol/kg for 1 week	Very short study period	Low	B
Gawor et al., 2023 [95]	Dog	Water additive with pomegranate	Daily water additive	Plaque and calculus accumulation, Gingival Bleeding Index (GBI)	Beneficial	Water additive with pomegranate can reduce dental deposit accumulation and improve gingival health	Not specifically a diet additive	Low	C
Hassan et al., 2019 [96]	Dog	Herbal supplements	Garlic one tablet orally + (1.5 g/kg/day of Fenugreek seeds powder + 2 g/day of Nigella sativa seeds	Diabetes mellitus markers, liver function	Beneficial	Herbal therapy improved outcomes	Short study design and relatively small control group	Low	B
Reichling et al., 2004 [97]	Dog	Boswellia serrata resin extract	400 mg/10 kg body weight once daily	Clinical signs of osteoarthritis (lameness, pain, stiff gait)	Beneficial	Standardized Boswellia preparation can be recommended as herbal dietary supplement for symptomatic support in canine osteoarthritic disease	Open study design, no control group. Among all dogs, 5 had brief, reversible episodes of diarrhea and flatulence, but only one case was possibly related to the supplement	High	C
Reichling et al., 2003 [98]	Dog	Echinacea purpurea root powder	1.0 g/10 kg body weight once daily	Clinical symptoms of respiratory tract infections	Beneficial	Echinacea preparation can be recommended as well tolerated alternative treatment of canine upper respiratory tract infections	Open study with no control group; two adverse events were recorded—one case of kennel cough exacerbation and another of severe coughing not improved during the study, authors stated these were unrelated to intervention	High	C
Stokes et al., 2017 [99]	Cat	Clindamycin and synbiotic	75 mg clindamycin + 2 synbiotic capsules	Fecal score, food consumption, vomiting	Beneficial	Improved treatment completion and reduced vomiting	Limited to healthy research cats; short study duration; 4 cats (50%) in period 1 placebo group discontinued due to vomiting on 3 consecutive days, including 1 with concurrent hematemesis. In period 2, 1 in the placebo and 1 in the synbiotic group were withdrawn (the former due to vomiting, the latter due to weight loss)	Low	B
Hart et al., 2012 [100]	Cat	Multi-strain synbiotic	Proviable-DC (5B CFU/day)	Stool quality/diarrhea	Beneficial	Safe, improved chronic diarrhea in most	Open label, no placebo, subjective scoring; short study duration. One cat was excluded for refusing a meal containing the synbiotic; no other adverse events were reported in any participant	High	C
Lee et al., 2019 [101]	Dog	Sodium tripolyphosphate (food-grade additive)	750 mg/L (minimum inhibitory concentration)	Antimicrobial efficacy against periodontal pathogens, biofilm formation	Beneficial	Strong antimicrobial effects against Porphyromonas species; reduced biofilm formation at sub-MIC levels	Primarily in vitro study; limited clinical trial data	Low	F
Levine et al., 2016 [102]	Dog	Plant extracts (green tea, turmeric, rosemary)	3.1–6.3 μg/mL in culture media	Cancer cell proliferation inhibition, cytotoxicity	Beneficial	No cytotoxicity at effective concentrations; anti-proliferative effects on cancer cells	In vitro study only; no direct animal feeding trials	Low	F
Aquilina et al., 2012 [103]	Dog	Beta-carotene (vitamin A precursor)	Up to maximum dietary levels with <100 mg/kg triphenylphosphine oxide	Toxicological parameters, plasma/serum levels, adverse effects	Neutral	No maximum dietary limit necessary assuming triphenylphosphine oxide contamination <100 mg/kg	Regulatory assessment based on multiple study compilations; impurity concerns	Low	D
Anthony et al., 2021 [104]	Dog	Alpha-lipoic acid	2.7–4.94 mg/kg body weight/day	Antioxidant capacity, safety parameters	Beneficial	Alpha-lipoic acid is well tolerated and improves antioxidant capacity in dogs	Review article, not primary research	Low	D

**Table 2:** Assessment of preservative studies.

Study ID: [Unique Identifier]	Species: [Dog/Cat/Mixed]	Preservative Type: [Specific Compounds]	Exposure Level: [Dose/Concentration]	Outcomes: [Primary/Secondary Endpoints]	Effect Direction: [Harmful/Beneficial/Neutral]	Key Findings: [Brief Summary]	Limitations: [Major Concerns]	Risk of Bias [Low/High]	Quality Grade [A, B, C, D, F]
Steffanutti et al., 2024 [106]	Dog	Spirulina	None up to 2 g/kg of BW for 12 weeks.	Antioxidant capacity and Serum lipids	Neutral	Serum triglycerides decreased significantly from T0 to T1 in the Spirulina group (*p* < 0.0001) but not in the placebo group (*p* = 0.28).	Narrow scope, only focusing on oxidative stress	Low	B
Minieri et al., 2024 [107]	Dog	Oil-free olive pulp flour	None up to 11.5 mg/kg per day.	Oxidative blood markers, d-ROMS	Neutral	Throughout the entire experimental trial, Group B dogs showed no adverse effects from the olive powder (e.g., loss of appetite, diarrhea, vomiting, or any other potential issues).	Narrow scope, only focusing on oxidative stress	Low	B
Chou et al., 2016 [108]	Dog	Antimicrobial cranberry extract	None up to 1 g/25 kg body weight.	Bacteriostasis assay	Neutral	Cranberry extract prevented development of a UTI and prevented E coli adherence to MDCK cells.	Small sample size; cranberry extract decreased E coli adherence to MDCK cells but did not inhibit bacterial growth	Low	B
Rhouma et al., 2013 [109]	Dog	Antioxidant Vitamin E	None at 0.044 mL/kg body weight.	Prostoglandins, Nitrogen oxide, Interleukin 1	Beneficial	Vitamin E group showed improved lesions and inflammatory markers.	Narrow scope, 1 dose, not directly added to diet	Low	C
Allison et al., 2000 [110]	Cat	Antioxidant bioflavanoid	None up to 10 mg/day. Bioflavanoid decreased Heinz body formation but not methemoglobinemia.	Packed cell volume, percentage of erythrocytes with Heinz bodies, blood methemoglobin concentration, and blood reduced and oxidized glutathione concentrations	Neutral	Groups 2 and 3; 15 per group) exhibited transient cyanosis and lethargy during the first 12 h after acetaminophen administration; no adverse effects were associated with antioxidant administration alone.	Short study duration; 1 dose; not directly added to diet	Low	C
Hall et al., 2016 [111]	Dog	Antioxidants acetate tocopherol-α and Lipoic acid	None up to 955.18 IU/kg tocopherols or 100 mg/kg Lipoic acid for 3 months. Bioactive ingredients led to reduced markers of muscle and collagen breakdown.	Glomular filtration rate and serum metabolites	Beneficial	Six dogs were removed during the study for unrelated health issues (renal failure, heart failure, hemangiosarcoma, and liver failure), but these were not considered adverse effects of the experimental diets.	Variable isolation was low	High	B
Hong et al., 2024 [112]	Dog	Antioxidants Oligo-Fucoidan, Fucoxanthin, and L-Carnitine	None at 125 mg of oligo-fucoidan, 125 mg of high-soluble fucoxanthin, and 50 mg of L-carnitine per 5 kg of body weight. Seven dogs perished due to unrelated causes.	Vital signs, serum chemistry, and blood urea nitrogen (BUN), creatinine (CREA), calcium, inorganic phosphate (IP), and electrolytes	Beneficial	The OFL compounds showed a reno-protective effect, consistent with previous animal studies. Seven dogs perished due to unrelated causes.	Only 1 dose for intervention variable	Low	B
Li 2024 [113]	Dog	Antioxidant potential of black soldier fly protein hydrolysate	None up to 15% of diet for 33 days. Increased BSFP significantly decreased MDA but 10/15% showed increase over 5%.	Serum chemistry, Inflammatory cytokines, and antioxidant capacity	Beneficial	Supplementation with BSFPs had no negative influence on final body weight and average daily feed intake; decreased inflammatory markers, increased plasma calcium, and increased antioxidant enzyme activity.	Relatively short duration and control group	Low	A
Kusaba and Arai 2024 [114]	Dog	Antioxidant shiitake mushroom powder	None at 800 mg/kg body weight/day.	Serum chemistry, superoxide dismutase, and body condition score	Beneficial	Plasma total cholesterol concentrations decreased and superoxide dismutase activity and leukocyte sirtuin1 mRNA expression increased significantly in the dogs that received the supplement.	Short study duration and low sample size	Low	C
Majeed and Mahmood 2023 [115]	Dog	Ginger ethanolic extract, chitosan nanoparticles, and ginger ethanolic extract-loaded chitosan nanoparticles	None at 1.7 mg/kg body weight for 45 days.	Pancreatic tissue biopsy, DNA damage, Atomic Force Microscopy	Neutral	Significant DNA damage reduction.	Low control group size	Low	B
Stefanutti et al., 2023 [116]	Dog	Spirulina	0.06 to 0.19 for small-sized dogs, from 0.05 to 0.15 for medium-sized dogs, and from 0.04 to 0.12 for large-sized dogs (g/kg BW).	Vomiting, diarrhea, skin and coat, behavior	Neutral	Dogs: 8.3% (5 out of 60) experienced mild adverse effects including vomiting or diarrhea, leading to discontinuation in isolated cases.	No control group	Low	B
Stefanutti et al., 2023 [116]	Cat	Spirulina	0.08 to 0.25 (g/kg BW) for cats.	Vomiting, diarrhea, skin and coat, behavior	Neutral	Cats: 1 case of auricular dermatitis and a few instances of vomiting were reported early in dosing escalation; no severe or persistent reactions occurred.	No control group	Low	B
Mo et al., 2023 [117]	Cat	Chitosan	None up to 2000 (H-CS) mg/kg chitosan for a period of 60 days. Chitosan reduced MDA, improved SOD concentrations, and reduced water in stool.	Hematological analyses, fecal microbiota, serum anti-oxidative condition, serum intestinal barrier function, serum cytokines	Beneficial	Dietary chitosan supplementation impacted SCFA production-related bacteria, which ameliorated intestinal barrier dysfunction and intestinal health of cats.	No clear limitations	Low	A
He et al., 2023 [118]	Cat	Antimicrobial peptide from chicken intestines, cultured by Bacillus subtilis	None at 0.3% per diet.	Serum biochemistry, fecal microbiota, inflammatory markers	Beneficial	No adverse reactions were reported. Diarrhea rates were actually reduced in the group supplemented with AMPs, with no negative effects documented throughout the study.	Only 1 dose for intervention variable	Low	B
Gianetto et al., 2022 [119]	Dog	Antioxidant Sylimarin	None at 1 g/10 kg body weight.	Hematological analyses, oxidant/antioxidant activity, and liver ultrasound	Neutral	All animals tolerated silymarin supplementation well, with no gastrointestinal side effects observed.	Only 1 dose for intervention variable	Low	B
Sun et al., 2022 [120]	Cat	Maillard reaction products	None at 3% of diet. MRPs in this study could significantly increase the scavenging ability of OH and DPPH radicals and the chelating ability of Fe^2+^.	Activity of free radical scavenging, volatile compound analysis	Neutral	No adverse reactions were described; the study reports that acceptability and feeding rates improved with attractant addition, and there is no mention of negative effects or withdrawals.	Very short duration; narrow scope with lack of safety assessment	Low	C
Bampidis et al., 2022 [121]	Dog	Antimicrobial Bifidobacterum	2.9 × 10^9^ cfu/kg of feed.	Serum chemistry, antimicrobial resistance, feces quality.	Neutral	To support the efficacy of this type of additive, when a claim of improving animal welfare and resilience to stress factors is given, positive changes in both relevant physiological and behavioral parameters are needed.	N/A	Low	A
Sechi et al., 2022 [122]	Dog	Antioxidants per kg of diet: Grifola frondosa: 270 mg/kg, Curcuma longa: 102 mg/kg, Carica papaya: 135 mg/kg Punica granatum: 70 mg/kg, Aloe vera: 135 mg/kg, Polygonum cuspidatum: 7 mg/kg, Solanum lycopersicum: 250 mg/kg, Vitis vinifera: 24 mg/kg, Rosmarinus officinalis: 15.6 mg/kg	None at 1 g/kg of diet for 6 months.	Hematological analyses, biochemical properties, and blood fatty acid profile	Neutral	Arachidonic acid was lower in the CH-AOX and FH-AOX groups; FH led to a better FA profile than that of the CH diet, while CH-AOX and FH-AOX improved the FA profile regardless of the basal diet.	Small sample size; only 1 dose for intervention variable	Low	B
Park et al., 2022 [123]	Dog	Antioxidants: grape seed extract, Vitamin E, alpha-lipoic acid, Vitamin C, Astaxanthin, curcuminoid, etc.	None over 5-year period, dosage unknown.	Cataract progression analysis	Beneficial	No adverse reactions reported for Ocu-GLO or Meni-One Eye RC supplementation; both Ocu-GLO (hazard ratio = 0.265, *p* = 0.026) and Meni-One (hazard ratio = 0.246, *p* = 0.005) significantly delayed the progression of immature cataracts compared to the control group.	Narrow scope and lack of safety assessment	Low	B
Suartha et al., 2022 [124]	Dog	Antioxidant/antimicrobial Trigona honey	None at 5 mL/dog/day for 5 weeks.	Hematological analysis and serum biochemistry	Neutral	The study specifically concludes that Trigona honey is safe—no adverse physiological effects occurred during treatment.	Very small control group	Low	B
de Santiago et al., 2021 [125]	Dog	Antioxidants rosemary, green tea, citrus pulp, and vitamin C	None over 60-day period, dosage unknown.	Priuritis score	Beneficial	Zero adverse reactions were reported. Study completion rate was 100%, and there were no reports of negative effects associated with either diet.	Narrow scope and limited safety assessment	Low	B
Bampidis et al., 2021 [126]	Cat	Antioxidant Butylated Hydroxy-Anisole BHA	None up to 750 mg/kg of diet.	Hematological analysis, serum biochemistry, feces quality	Neutral	Feed intake was decreased at highest dosage but likely due to palatability; only one case of vomiting was reported in a cat in the control group.	N/A	Low	A
EFSA 2011 [127]	Dog	Butylated hydroxyanisole	None up to 325 mg/kg bw/day.	Toxicity	Neutral	Dose-related retardation of growth was reported; the data showed no histopathological effects after 6 months of exposure to BHA in the diet in the stomach, esophagus, duodenum, or liver of beagle dogs; overall, studies performed on dogs did not show any proliferative changes in the stomach at dose levels up to 325 mg/kg bw/day, the highest dose tested.	N/A	Low	A
Anthony et al., 2021 [128]	Dog	Alpha-lipoic acid	None at 300 ppm of alpha-lipoic acid added for 6 months. One adverse reaction which was unrelated to study. GI complications were unrelated as they resolved while diets continued.	Serum chemistry, complete blood count, urinalysis, physical examination	Neutral	One dog in a high-dose group died due to anaplastic sarcoma, determined not to be study-related, and all other mild events (e.g., isolated cases of vomiting or cough) resolved without intervention or were considered unrelated to the diet.	Narrow scope and limited safety assessment	Low	B
Meineri et al., 2021 [129]	Dog	Probiotics (Lactobacillus acidophilus), prebiotics (fructoligosaccharides), and antioxidants (Olea Europaea extract)	None up to 40 mg/day for 90 days.	Blood pressure, body weight, hematological analysis, blood biochemistry, c-reactive protein, urinary protein/creatinine	Beneficial	Significant improvement of the protein plasmatic level and a decrease in blood phosphorus, systolic pressure, BUN, proteinuria, and urine protein-to-creatinine ratio throughout the trial in the TG compared to the CG.	Synergistics focus rather than isolation of variables	Low	B
Sechi et al., 2017 [130]	Dog	Antioxidant nutraceuticals: Punica granatum 457 mg/kg, Valeriana officinalis 260 mg/kg, Rosmarinus officinalis 0.44 mg/kg, Tilia species 635 mg/kg, Crataegus oxyacantha 392 mg/kg, l-Theanine 310 mg/kg, l -Tryptophan 329 mg/kg	2.38 g/kg of diet.	Biological antioxidant potential, neurotransmitter serum concentrations, hematological analysis, endorphins	Beneficial	The study specifically states that the nutraceutical diet was “highly tolerated without any adverse effects”; stress markers decreased and reactive oxygen species decreased significantly.	Relatively narrow scope and low isolation of variables	Low	B
Superchi et al., 2016 [131]	Dog	Antimicrobial chabazite/phillipsite	None at 5 g per day for 29 days. No changes to fecal score and body weight.	TBARS, fecal characteristics, hematological analysis, Nitric oxide	Beneficial	A reduction of 40% in thiobarbituric acid reactive substances (TBARS) levels was observed in Z compared to the C group (*p* < 0.05); all dogs completed the study and consumed all rations, and no negative effects were reported with chabazite-phillipsite supplementation.	Narrow scope; short study duration	Low	B
Wang et al., 2016 [132]	Dog	Antioxidant blend: lutein 20 mg, zeaxanthin 5 mg, β-carotene 20 mg, astaxanthin 5 mg, vitamin C 180 mg, and vitamin E 336 mg	None at 0.57 g twice per day.	Scotopic and Photopic Electroretinography	Beneficial	All ERG a-wave and b-wave amplitudes increased with antioxidant group; No adverse reactions reported. All dogs completed the study in good health, with body weight maintained throughout the study.	Small sample size; Narrow scope with limited food safety parameters	Low	C
Hall et al., 2016 [133]	Cat	Antioxidants α-tocopherol acetate (>900 IU/kg, as fed), 200 mg/kg vitamin C, and 300 mg/kg L-carnitine	None at 1.16 g/kg of diet for 6 months.	Serum and urine analysis	Neutral	The study protocol included removal of cats for any adverse events, but all 80 cats completed the trial, and no withdrawals due to negative effects were noted.	Lack of control in the owners’ choice group; narrow scope and limited food safety assessment	High	C
Valentina et al., 2015 [134]	Dog	Antioxidants rose-hip extract 500 mg and grapeseed extract 100 mg	None at 0.6 g/kg of diet.	Reactive oxygen metabolites, MDA, Ferric reducing ability of plasma assay	Beneficial	All dogs completed diet treatments and exercise regimens according to protocol, and no negative physiological effects are noted in the text.	Narrow scope with limited food safety parameters	Low	B
Pan et al., 2012 [135]	Cat	Antioxidants: alpha Tocopherol acetate 550 mg/kg, Vitamin C 80 mg/kg, Selenium 1 g/kg	None at 1.63 g/kg of diet.	DNMP cognitive test, body weight, serum fatty acid profile, and antioxidant status	Beneficial	Baseline cognitive ability improved but fasting fatty acid profile/antioxidant capacity did not differ.	Narrow scope with limited food safety parameters; only 1 intervention variable	Low	C
EFSA 2012 [136]	Dog	Antimicrobial potassium sorbate	4% of diet.	Hemoglobin concentrations, histological examinations, body weight	Neutral	The FEEDAP Panel concluded that potassium sorbate is safe for both dogs and cats at a maximum level of 5000 mg/kg semi-moist complete feed.	Small sample size	Low	B
EFSA 2012 [136]	Cat	Antimicrobial potassium sorbate	2% of diet.	Hemoglobin concentrations, histological examinations, body weight	Neutral	The FEEDAP Panel concluded that potassium sorbate is safe for both dogs and cats at a maximum level of 5000 mg/kg semi-moist complete feed	Small sample size, relatively short duration	Low	B
Raila et al., 2011 [137]	Dog	Antioxidant Tocotrienols	None at 40 mg/kg body weight of a tocotrienol-rich fraction (TRF).	Plasma total antioxidant status and tocotrienols in chylomicrons	Neutral	The increase in antioxidant capacity suggests a potential beneficial role of TCT supplementation in the prevention or treatment of several diseases in dogs.	Very small sample size, short duration, and lacking multiple doses	Low	C
Pop et al., 2010 [138]	Dog	Antioxidant alpha tocopherol acetate 21 mg/kg, ascorbic acid 1.6 mg/kg, acetyl-l-carnitine 5.2 mg/kg, and dl-α-lipoic acid 2.6 mg/kg	None at 0.3 g/10 kg body weight for 2.69 years.	Aβ neuropathology in plaques, biochemically extractable Aβ40 and Aβ42 species, soluble oligomeric forms of Aβ, and various proteins in the β-amyloid precursor protein (APP) processing pathway	Neutral	No adverse reactions or negative effects from antioxidant diets or enrichment were reported; all surviving dogs completed the study protocol, and interventions were well tolerated.	Narrow scope and limited safety assessment	Low	B
Opii et al., 2006 [139]	Dog	Antioxidants Vitamin E 1000 ppm, L-carnitine 250 ppm, Vitamin C 80 ppm, DL alpha lipoic acid 120 ppm	None at 1.45 g/kg of diet for 2.8 years.	Protein carbonyls, 3-nitrotyrosine, HNE, SOD, GST, brain samples, and cognitive testing	Neutral	No adverse reactions attributed to interventions were reported; only incidental unrelated events (e.g., liver degeneration, pancreatitis, heart failure, anorexia, abscess) were noted. All deaths or removals were deemed unrelated to antioxidant or enrichment treatments.	Narrow scope and limited safety assessment	Low	B
Waters et al., 2003 [140]	Dog	Antioxidant Selenium	None up to 6 μg/kg body weight per day for 7 months. Daily supplementation of selenium yeast was associated with a reduction in genotoxic damage to the canine prostate.	DNA damage, prostatic epithelial cell apoptosis, Glutathione peroxidase	Beneficial	No adverse reactions were reported during the study. All dogs remained healthy and completed the trial according to protocol.	Narrow scope and limited safety assessment	Low	B
Liebert 1988 [141]	Dog	Antimicrobials sorbic acid and potassium sorbate	None at 10% of the diet in subchronic studies.	Mutagenic effects using the Ames test, genetic recombination tests, reversion assays, rec assays, tests for chromosomal aberrations, sister chromatid exchanges, and gene mutations.	Neutral	No adverse effects attributable to potassium sorbate or sorbic acid were found in dogs after 3 months at dietary concentrations up to 8% sorbic acid or 1–2% potassium sorbate.	Low sample size	Low	B
Baskin et al., 2000 [142]	Dog	Antioxidants alpha tocopherol acetate 400 UI/kg, β-carotene 3 mg/kg, and lutein 20 mg/kg orally per day	None up to 0.317 g/kg of diet for 1 month. Supplementation decreased DNA oxidation and increased resistance of lipoprotein particles to in vitro oxidation.	Plasma antioxidant concentrations, oxidative DNA damage, membrane lipids, plasma lipoproteins	Beneficial	No dogs were withdrawn from the study due to injury, fatigue, or other medical problems. There is no report of adverse reactions in any group.	Narrow scope and limited safety assessment	Low	B
Longobardi 2024 [143]	Cat	Green tea extract	None up to 400 µg/mL.	Apoptotic markers, ROS, antioxidant capacity	N/A	No adverse reactions (cytotoxic effects) were observed at concentrations up to 200 µg/mL. Significant reduction in cell viability occurred only at 400 µg/mL, which was excluded from subsequent analyses (i.e., no adverse reactions were reported under the experimental conditions used).	Infected cell study derived from cats, not directly applicable	Low	D
Zhang 2024 [144]	Dog	Limosilactobacillus reuteri	None up to 2 × 10^9^ CFU/mL per day.	Organ index, antioxidant capacity, genome sequence of microbiome	N/A	L. reuteri LRA7 has good probiotic potential and is safe.	In vitro study on antimicrobial activity using LRA7 strain	Low	D
Scahill 2023 [145]	Dog	Antimicrobials Amoxicillin-clavulanic acid and metronidazole	Antimicrobials did not reduce duration of occurrence of diarrhea but did not worsen symptoms. Probiotics and synbiotics had no clinically relevant effect on diarrhea duration. Prebiotics had a small favorable effect in a single study.	Acute diarrhea	N/A	N/A	Review article	Low	F
Moritz 2024 [146]	Dog	Antioxidant and antimicrobial luteolin, quercetin, and grape seed extract oligomeric proanthocyanidins	None at 12.5, 25, and 50 μg/mL of Quercetin. Luteolin exhibited cytotoxic effects at all doses (12.5, 25, and 50 μg/mL) (*p* < 0.001). Grape seed extract had pro-inflammatory effects at 25 μg/mL but not at 50 μg/mL.	Canine white blood cells: Reactive oxygen species, cellular metabolic activity, and tumor necrosis factor alpha	N/A	N/A	In vitro study using cell cultures	Low	D

**Table 3:** (**a**) Nutritional assessment of human-grade vs. feed-grade ingredients. (**b**) Safety assessment of human-grade vs. feed-grade ingredients.

(a)										
Reference	Diet/Ingredients	Ingredient Type	Category	Nutritional Effects	Digestibility Effect	Outcome Hierarchy [Primary, Secondary, Tertiary]	Effect Direction [Beneficial, Neutral, Harmful]	Risk of Bias [Low/High]	Quality of Evidence	Limitations/Key Findings
Roberts 2023 [148]	Life Protection Formula Chicken and Brown Rice, Blue Buffalo, Wilton, CT. Ingredients: deboned chicken, chicken meal, brown rice, barley, oatmeal, pea starch, flaxseed, chicken fat, dried tomato pomace, natural flavor, peas, pea protein, salt, potassium chloride, dehydrated alfalfa meal, potatoes, dried chicory root, pea fiber, alfalfa nutrient concentrate, calcium carbonate, choline chloride, DL-methionine, preserved with mixed tocopherols, dicalcium phosphate, sweet potatoes, carrots, garlic, zinc amino acid chelate, zinc sulfate, vegetable juice for color, ferrous sulfate, vitamin E supplement, iron amino acid chelate, blueberries, cranberries, barley grass, parsley, turmeric, dried kelp, yucca schidigera extract, niacin (vitamin B3), glucosamine hydrochloride, calcium pantothenate (vitamin B5), copper sulfate, biotin (vitamin B7), L-ascorbyl-2-polyphosphate, L-lysine, L-carnitine, vitamin A supplement, copper amino acid chelate, manganese sulfate, taurine, manganese amino acid chelate, thiamine mononitrate (vitamin B1), riboflavin (vitamin B2), vitamin D3 supplement, vitamin B12 supplement, pyridoxine hydrochloride (vitamin B6), calcium iodate, dried yeast, dried Enterococcus faecium fermentation extract, dried Trichoderma longibrachiatum fermentation extract, dried Bacillus subtilis fermentation extract, folic acid (vitamin B9), sodium selenite, oil of rosemary	Feed-grade	Nutrition	Protein 26.69% DM, Methionine/Cysteine 71% above minimum	True Metabolizable Energy 3.99 kcal/g (baseline), Energy Efficiency 77.3% TMEn/GE (baseline)	Primary	Beneficial	High	B	True metabolizable energy was higher for Human-grade diets than feed-grade control. Human-grade diets contained higher taurine and feed-grade diets contained higher methionine/cysteine. Human-grade recipes also showed higher protein; Company funding, no blinding mentioned, rooster model limitations
	BC: The Roost, Bramble Inc., New York, NY. Ingredients: organic pea protein, long grain brown rice, potato, garbanzo beans, carrots, organic sunflower oil, peas, butternut squash, blueberries, malt extract, potato starch, nutrient mix [choline chloride, potassium chloride, L-methionine, tricalcium phosphate, taurine], vitamins [D-calcium pantothenate, riboflavin, niacin, vitamin B12, vitamin A acetate, vitamin E supplement, folic acid, thiamine mononitrate, pyridoxine hydrochloride, vitamin D2 supplement], trace minerals [zinc proteinate, iron proteinate, copper proteinate, manganese proteinate, calcium iodate, selenium yeast], nutritional yeast, tricalcium phosphate, potassium chloride, sodium phosphate, magnesium, salt.	Human-grade		Protein 32.50% DM (+21.8% increase), Lysine 8% lower than control, Histidine 15.8% lower than control, Threonine 8.7% lower than control, Methionine/Cysteine 48% above minimum	True Metabolizable Energy 4.55 kcal/g (+14.0% increase), Energy Efficiency 80.4% TMEn/GE (+4.0% efficiency)					
	BR: The Cowbell, Bramble Inc., New York, NY. Ingredients: organic pea protein, lentil, sweet potato, carrots, organic sunflower oil, organic flax oil, peas, apples, malt extract, potato starch, nutrient mix [choline chloride, potassium chloride, L-methionine, tricalcium phosphate, taurine], vitamins [D-calcium pantothenate, riboflavin, niacin, vitamin B12, vitamin A acetate, vitamin E supplement, folic acid, thiamine mononitrate, pyridoxine hydrochloride, vitamin D2 supplement], trace minerals [zinc proteinate, iron proteinate, copper proteinate, manganese proteinate, calcium iodate, selenium yeast], nutritional yeast, caramel color, tricalcium phosphate, potassium chloride, sodium phosphate, magnesium, salt.	Human-grade		Protein 34.20% DM (+28.1% increase), Methionine/Cysteine 60% above minimum, Taurine +41% vs control	True Metabolizable Energy 4.64 kcal/g (+16.3% increase), Energy Efficiency 84.7% TMEn/GE (+9.6% efficiency)					
Geary et al. 2022 [150]	Blue Buffalo: Life Protection Formula Chicken and Brown Rice Recipe	Feed-grade	Nutrition	Fecal pH Increase +0.27 (more alkaline), Transepidermal Water Loss −2.88 g/h/m^2^ (moderate improvement), Hair surface score −0.27 (improvement), Superoxide Dismutase +0.21 fold change, TNF-α Expression +0.20 fold change, COX-2 Expression +0.05 fold change, serum cholesterol Increase +21.00 mg/dL, serum protein −0.16 g/dL, caloric efficiency −155.54 kcal/day decrease with maintained body weight,	N/A	Tertiary	Beneficial	High	B	Human-grade diet produced higher digestive efficiency, enhanced skin barrier function, improved serum cholesterol and protein, and large shift in gut microbiome. (Further studies are needed to classify microbiome shift as positive or negative); Non-randomized baseline differences, company funding, lack of true control
	Just Food for Dogs: Chicken and White Rice Recipe	Human-grade	Nutrition	Fecal pH Decrease −1.00 (more acidic—beneficial), Transepidermal Water Loss Decrease −6.98 g/h/m^2^ (significant improvement), hair surface score +0.28 (surface damage noted), superoxide dismutase +0.21 fold change, TNF-α Expression +0.70 fold change, COX-2 Expression +0.43 fold change, serum cholesterol Decrease −34.80 mg/dL, serum protein +0.31 g/dL, KEGG 68 pathways increased and 98 pathways decreased, caloric efficiency +58.16 kcal/day increase						
Oba et al. 2019 [152]	Beef & Russet Potato (ingredients: ground beef, russet potatoes, sweet potatoes, green beans, carrots, safflower oil, beef liver, green peas, apples, Icelandic premium EPA and DHA, natural calcium, phosphorus amino acid chelate, magnesium bisglycinate chelate, taurine, choline chloride, natural kelp, vitamin E, biotin, selenium amino acid chelate, manganese bisglycinate chelate, zinc oxide, vitamin D3, vitamin B1, riboflavin).	Human-grade	Nutrition	N/A	Dry Matter Digestibility 74%, Organic Matter Digestibility 81.9%	Primary	Neutral	High	C	Company funding, no blinding, rooster assay (not in-dog validation)
	Chicken & White Rice (ingredients: chicken thigh, long grain white rice, spinach, carrots, apples, chicken gizzard, chicken liver, Icelandic premium EPA and DHA, calcium pyrophosphate, natural calcium, choline bitartrate, natural kelp, magnesium bisglycinate chelate, iron bisglycinate chelate, copper bisglycinate chelate, vitamin D3, vitamin B12, riboflavin)				Dry Matter Digestibility 82.3%, Organic Matter Digestibility 89.2%					
	Fish & Sweet Potato (ingredients: Pacific cod, sweet potatoes, russet potatoes, green beans, broccoli, safflower oil, natural calcium, phosphorus amino acid chelate, natural kelp, choline chloride, vitamin E, iron bisglycinate chelate, zinc oxide, biotin, copper citrate, riboflavin, vitamin B12)				Dry Matter Digestibility 67.2%, Organic Matter Digestibility 76.4%					
	Lamb & Brown Rice (ingredients: ground lamb, long grain brown rice, cauliflower, carrots, lamb liver, spinach, blueberries, safflower oil, Icelandic premium EPA and DHA, natural calcium, phosphorus amino acid chelate, choline bitartrate, potassium chloride, natural kelp, sodium chloride, vitamin E, iron citrate, selenium amino acid chelate, zinc oxide, vitamin D3, riboflavin).				Dry Matter Digestibility 81%, Organic Matter Digestibility 87.3%					
	Turkey & Whole Wheat Macaroni (ingredients: ground turkey, whole wheat macaroni, broccoli, zucchini, carrots, turkey liver, cranberries, premium EPA and DHA, natural calcium, phosphorus amino acid chelate, choline bitartrate, potassium chloride, natural kelp, sodium chloride, taurine, vitamin E, magnesium bisglycinate chelate, zinc oxide, copper bisglycinate chelate, manganese gluconate, vitamin D3, riboflavin, vitamin B12, vitamin B1).				Dry Matter Digestibility 78%, Organic Matter Digestibility 81.9%					
	Venison & Squash (ingredients: venison, butternut squash, sweet potatoes, brussel sprouts, cranberries, safflower oil, premium EPA and DHA, natural calcium, phosphorus amino acid chelate, choline bitartrate, potassium chloride, natural kelp, sodium chloride, taurine, vitamin E, magnesium bisglycinate chelate, zinc oxide, copper bisglycinate chelate, manganese gluconate, vitamin D3, riboflavin, vitamin B12, vitamin B1).				Dry Matter Digestibility 67.6%, Organic Matter Digestibility 74.1%					
Do et al. 2021 [149]	Chicken and Brown Rice Recipe (extruded; Blue Buffalo)	Feed-grade	Nutrition	Fecal score: 2.63, total SCFA 490.64 µmol/g DM, total BCFA 15.57 µmol/g DM, ammonia 98.91 µmol/g DM	Dry Matter Digestibility 81.47%, Protein Digestibility 83.396%, Hydrolyzed Fat Digestibility 93.33%	Primary	Beneficial	High	B	Company funding, limited randomization details, no blinding
	Roasted Meals Tender Chicken Recipe (fresh; Freshpet)	Human-grade		Fecal score: 2.75, total SCFA 490.64 µmol/g DM, total BCFA 15.57 µmol/g DM, ammonia 98.91 µmol/g DM	Dry Matter Digestibility +6.96%, Protein Digestibility +12.26%, Hydrolyzed Fat Digestibility +3.8%					
	Beef & Russet Potato Recipe (HG beef; JustFoodForDogs)	Human-grade		Fecal score: 2.75, total SCFA 490.64 µmol/g DM, total BCFA 15.57 µmol/g DM, ammonia 98.91 µmol/g DM	Dry Matter Digestibility +11.5%, Protein Digestibility +11.31%, Hydrolyzed Fat Digestibility +5.61%					
	Chicken & White Rice Recipe (HG chicken; JustFoodForDogs)	Human-grade		Fecal score: 2.75, total SCFA 490.64 µmol/g DM, total BCFA 15.57 µmol/g DM, ammonia 98.91 µmol/g DM	Dry Matter Digestibility +11.34%, Protein Digestibility +11.25%, Hydrolyzed Fat Digestibility +4.46%					
**(b)**										
**Reference**	**Ingredient**	**Ingredient Type**	**Outcome**	**Measured Level vs. Human-Grade Regulatory Limit**	**Safety Assessment**	**Outcome Hierarchy [Primary, Secondary, Tertiary]**	**Effect Direction [Beneficial, Neutral, Harmful]**	**Risk of Bias** **[Low/High]**	**Quality of Evidence**	**Limitations/Key Findings**
Spears et al., 2017 [153]	Whole Corn	Feed-grade	Chromium 0.026 (0.008–0.054)	Less than 3% of FDA 1.0 mg/kg limit	Suitable for human food	Primary	Harmful	Low	B	No human-grade comparison; FDA standards utilized
	Whole Wheat	Feed-grade	Chromium 0.041 (0.029–0.062)	4.1% of FDA 1.0 mg/kg limit	Suitable for human food					
	Soybean Meal	Feed-grade	Chromium 0.208 (0.154–0.286)	20.8% of FDA 1.0 mg/kg limit	Suitable for human food					
	Alfalfa Hay	Feed-grade	Chromium 0.522 (0.199–0.889)	52.2% of FDA 1.0 mg/kg limit	Suitable for human food					
	Beet Pulp	Feed-grade	Chromium 1.222 (0.776–1.451)	122% of FDA 1.0 mg/kg limit	Not suitable for human food					
	Feed Phosphates	Feed-grade	Chromium 135.0 (112.0–163.0)	135x higher than FDA 1.0 mg/kg limit	Not suitable for human food					
Tripathi et al., 2007 [154]	Human-food-grade maize (100%)	Human-grade	0 (no contamination) Aflatoxin B1	Compliant with FDA human-grade limits and feed-grade 20 μg/kg maximum	Suitable for human consumption	Primary	Harmful	Low	A	Feed-grade wheat showed increasing dose response for Aflatoxin B1 concentrations, posing a potential food safety risk
	Feed-grade damaged wheat (25%) + human-grade maize (75%)	Feed-grade/Human-grade	5.9 (μg/kg diet) Aflatoxin B1 Content	29.5% of FDA 20 μg/kg maximum	No suitable for human food					
	Feed-grade damaged wheat (50%) + human-grade maize (50%)	Feed-grade/Human-grade	11.8 (μg/kg diet) Aflatoxin B1 Content	59% of FDA 20 μg/kg maximum	No suitable for human food					
	Feed-grade damaged wheat (75%) + human-grade maize (25%)	Feed-grade/Human-grade	17.6 (μg/kg diet) Aflatoxin B1 Content	88% of FDA 20 μg/kg maximum	No suitable for human food					
	Feed-grade damaged wheat (100%)	Feed-grade	23.5 (μg/kg diet) Aflatoxin B1 Content	117.5% of FDA 20 μg/kg maximum	Not suitable for human food or pet food					

**Table 4:** Assessment of whole vs. processed ingredients.

Reference	Ingredient(s)	Processing Step	Degree of Processing	Nutritional Effects	Digestibility Effect	Effect Direction [Beneficial, Neutral, Harmful]	Outcome Hierarchy [Primary, Secondary, Tertiary]	Risk of Bias [Low/High]	Quality of Evidence	Limitations
Kunyanga et al. 2011 [161]	Vegetables	Cut/washed/Blanched at 100 C for 5 min	Intensive	No significant phenolic losses in most vegetables, aside from a 74% reduction in amaranth leaves and 43% in pumpkin leaves, significant reductions in FRAP values for most vegetables, significant losses in DPPH radical scavenging activity, no effect on α-glucosidase inhibition activity, complete loss of α-amylase inhibition in butternut and sweet potato.	Not assessed	Harmful	Tertiary	Low	C	No control or minimal processing comparison. Narrow scope.
		Cut/washed/Steamed at 90–95 °C for 5 min	Moderate	No significant changes in phenolic content aside from a 45% reduction in amaranth leaves and 14% increase in sweet potatoes. Preserved DPPH radical scavenging activity and FRAP with small losses in butternut and sweet potatoes. Maintained α-amylase and α-glucosidase inhibition.	Not assessed	Neutral				
	Cereals, Legumes, and Oil Seeds	Washed/soaked/Blanched at 90–95 °C for 120 min	Intensive	Significant reduction (35–79%) in total phenolic content in most cereals and legumes, preserved DPPH radical scavenging activity, maintained reducing power (FRAP), preserved α-amylase and α-glucosidase inhibition activities.	Not assessed	Harmful				
		Roasted at 150 °C for 30 min	Intensive	No significant reduction in total phenolic content, significant increase in groundnut phenolic content (12%), significant losses in DPPH radical scavenging activity in most samples (except finger millet, field bean, and sunflower seed), enhanced FRAP values in finger millet, sunflower seed, and amaranth grain due to Maillard product formation, no significant changes in α-amylase and α-glucosidase inhibition except an increase in finger millet and sunflower seeds.	Not assessed	Neutral				
Albarracin et al. 2019 [162]	Rice Flour	Soaking in 5.5 g/L lactic acid solution at 45 °C for 24 h	Moderate	Reduced protein content but no effect on available lysine content, increased oleic acid content, decreased myristic acid levels, enhanced monounsaturated fatty acids, 65% reduction in phytic acid, reduced free phenolics but 32% increase in bound phenolics.	Protein digestibility decreased from 82.65% (rough rice) to 81.63%	Neutral	Primary	Low	A	Variable effects such as increase in digestibility, decrease in nutrients, decrease in antinutrients.
		Germination for 24 h at 85 °C with 98% relative humidity	Moderate	Reduced protein content with 3.3% decrease in lysine, 37% increase in linolenic acid content, 17% lower palmitic acid, 30% reduction in phytic acid; highest free phenolic content (73.32 mg gallic acid/100 g).	Dramatically improved protein digestibility to 100.5%	Beneficial				
		Extruded brown rice at 160 C and 14% moisture	Intensive	11.4% reduction in available lysine, 3% decrease in total dietary fiber, 34% increase in IP4 and IP3 forms. More than 100% increase in antioxidant capacity.	Decreased protein digestibility from 89.06% to 79.32%	Harmful				
		Extruded soaked rice at 160 C and 16.5% moisture	Intensive	No significant effect on available lysine, 40% decrease in total dietary fiber, >50% reduction in inositol phosphates and 20% decrease in phytic acid. More than 100% increase in antioxidant capacity.	Reduced protein digestibility from 81.63% to 74.71%	Harmful				
		Extruded germinated rice at 175 °C and 14% moisture	Intensive	5.8% reduction in available lysine, 41% decrease in total dietary fiber, 70% total reduction in phytic acid. Highest antioxidant capacity and bound phenolic content but 10% decrease in free phenolics.	16% decrease in protein digestibility from 100.5% to 83.62%	Harmful				
Mittal et al. 2012 [163]	Chickpea Flour	Germination (2 days at 22 °C)	Moderate	3.46% reduction in phytic acid, 81.99% reduction in polyphenols, 93.25% reduction in tannins, 22.72% reduction in Saponins, 58.97% reduction in Oxalates, 39.76% reduction in trypsin inhibitor activity, 48.42% increase in linolenic acid, Fe: −18.96%, K: −2.10%.	Not assessed	Neutral	Tertiary	Low	B	No control or minimal processing comparison.
		Boiling (1:7 *w*/*v*, 10 min)	Moderate	12.34% reduction in phytic acid, 86.44% reduction in polyphenols, 93.07% reduction in tannins, 36.36% reduction in Saponins, 43.58% reduction in Oxalates, 38.41% reduction in trypsin inhibitor activity, linolenic acid eliminated, Fe: +56.89%, K: +28.6%	Not assessed	Neutral				
		Pressure cooking (15 psi, 1:2 *w*/*v*, 15 min)	Intensive	13.7% reduction in phytic acid, 87.71% reduction (maximum) in polyphenols, 93.97% reduction (maximum) in tannins, 4.55% reduction in Saponins, 71.79% reduction (maximum) in Oxalates, 50.21% reduction (maximum) in trypsin inhibitor activity, Vicillins reduced by 50.16%, slight decrease in all fatty acids, K: −47.13%, *p*: −4.11%.	Not assessed	Harmful				
		Roasting (120 °C, 15 min)	Intensive	3.46% reduction in phytic acid, 82.20% reduction in polyphenols, 80.10% reduction in tannins, 25.00% reduction in Saponins, 46.15% reduction in Oxalates, 24.56% reduction in trypsin inhibitor activity, Vicillins reduced by −83.68% (maximum), 4.76% increase in palmitic acid, K: +5.27%, *p*: +2.88%.	Not assessed	Neutral				
Haverkort et al. 2023 [164]	Potatoes	Boiled in skin	Moderate	Moisture +1.1%, −7.9% protein, +100.0% fat, −0.9% carbohydrates, −33.3% fiber, +10.6% potassium, −35.6% Vitamin C.	Not assessed	Harmful	Primary	Low	B	No control or minimal processing comparison.
		Boiled and peeled	Moderate	Moisture −8.6%, −7.9% protein, +20.0% fat, +13.6% carbohydrates, −42.2% fiber, −21.6% potassium, −53.6% Vitamin C.	Not assessed	Harmful				
		Baked in skin	Intensive	Moisture −4.8%, +36.8% protein, +0% fat, +18.9% carbohydrates, +37.8% fiber, +31.5% potassium, −12.4% Vitamin C.	Not assessed	Beneficial				
		Pan-fried	Intensive	Moisture −40.6%, +110.5% protein, +14,100.0% fat, +120.7% carbohydrates, +0% fiber, −96.4% potassium, −2.1% Vitamin C.	Not assessed	Neutral				
		Flour/dehydrated	Intensive	Moisture −91.9%, +292.1% protein, +520.0% fat, +376.5% carbohydrates, +397.8% fiber, +12.5% potassium, −41.2% Vitamin C.	Not assessed	Beneficial				
Singh et al. 2023 [165]	Peas, Lentils, and Beans (Pulse)	Extruded and milled pulse 15%	Intensive	Methionine: −12.8%, GSH: +33.3%, taurine: no change, MCH: lower vs. pulse 30/45. All cardiac parameters normal with no DCM indicators. Urea: higher vs. pulse 45. No adverse effects over 20 weeks.	Not assessed	Neutral	Primary	Low	C	No control or minimal processing comparison. Narrow scope.
		Extruded and milled pulse 30%	Intensive	Methionine: −10.6%, GSH: +16.7%, taurine: no change, MCH: higher vs. pulse 15, phosphorus: +8.2%, glucose: −10.0%, All cardiac parameters normal with no DCM indicators. No adverse effects over 20 weeks.	Not assessed	Neutral				
		Extruded and milled pulse 45%	Intensive	Methionine: −14.9% (lowest), GSH: +16.7%, taurine: no change, MCH: higher vs. pulse 15, phosphorus: +8.2%, creatinine: Lower, urea: lower vs. pulse 15. All cardiac parameters normal with no DCM indicators. No adverse effects over 20 weeks.	Not assessed	Neutral				
Kim et al. 2022 [166]	10% Whole Soybeans	Extrusion: 425 rpm, 101.58 °C die temp	Intensive	Decrease 3.5% protein, +42.9% fat, +172.0% trypsin inhibitor reduction, +16.4% Urease activity reduction.	Not assessed	Beneficial	Primary	Low	B	No control or minimal processing comparison.
	20% Whole Soybeans	Extrusion: 425 rpm, 100.72 °C die temp	Intensive	Decrease 8.3% protein, +85.7% fat, +212.0% trypsin inhibitor reduction, +41.8% Urease activity reduction.	Not assessed	Beneficial				
	30% Whole Soybeans	Extrusion: 425 rpm, 99.58 °C die temp	Intensive	Decrease 5.9% protein, +133.3% fat, +212.0% trypsin inhibitor reduction, +44.8% Urease activity reduction.	Not assessed	Beneficial				
Loader et al. 2021 [167]	Whole Unprocessed Black Bean	Whole unprocessed, ground into flour	Minimal	Fat 3.28 g/100 g, protein 21.3 g/100 g, carbohydrates 70 g/100 g, total fiber 26.8 g/100 g, insoluble fiber 21 g/100 g, soluble fiber 5.7 g/100 g, ash 4.9 g/100 g.	Not assessed	Beneficial	Primary	Low	B	Lacked moderate methods for dose response.
	Black Bean BB Boiled and Freeze-Dried BB (No Milling)	Overnight soak, boiled at 100 C for 1 h, freeze-dried	Intensive	−15.15% in fat, +2.3% protein, +3.6% carbohydrates, +16.4% total fiber, +10% insoluble fiber, +42.1% soluble fiber, −40.8% ash.	Not assessed	Neutral				
	Black Bean BFM (Boiled Fine Milled)	Overnight soak, boiled at 100 C for 1 h, freeze-dried, fine mill	Intensive	−16.8% in fat, +4.2% protein, +3.6% carbohydrate, −3.4% total fiber, −6.7% insoluble fiber, +10.5% soluble fiber, −46.9% ash.	Not assessed	Neutral				
	Black Bean BCM (Boiled Coarse Milled)	Overnight soak, boiled at 100 C for 1 h, freeze-dried, coarse mill	Intensive	−34.5% in fat, +4.2% protein, +4.4% carbohydrates, +23.9% total fiber, +26.2% insoluble fiber, +17.5% soluble fiber, −46.9% ash.	Not assessed	Neutral				
	Black Bean MFM (Micronized Fine Milled)	Infrared processing: 90 s at 100 °C, fine mill	Intensive	−9.5% fat, +1.9% protein, +1.6% carbohydrates, −22.4% total fiber, −28.1% insoluble fiber, −3.5% soluble fiber, −12.2% ash.	Not assessed	Harmful				
	Black Bean ExFM (Extruded Fine Milled)	High-temperature extrusion: 30–120 °C zones + fine mill	Intensive	−16.8% in fat, +1.9% protein, +2% carbohydrates, −25.7% total fiber, −36.2% insoluble fiber, +12.3% soluble fiber, −22.2% ash.	Not assessed	Harmful				
	Black Bean DhFM (Dehulled Fine Milled)	Dehulled, soaked, boiled at 100 °C for 30 min, freeze-dried and fine-milled	Intensive	−25.6% in fat, +2.8% protein, +5.9% carbohydrates, −8.2% total fiber, −10% insoluble fiber, −1.8% soluble fiber, −69.4% ash.	Not assessed	Harmful				
Alvarenga et al. 2018 [168]	WSD (Whole Sorghum) Whole Grain Replacement	Extrusion: 98.5 °C preconditioner, 319.7 rpm shaft speed	Moderate	Decrease 11.6% fat, decrease 2.8% starch.	Not assessed	Harmful	Primary	Low	C	No control or minimal processing comparison. Narrow scope.
	FLD (Sorghum Flour) Flour Fraction Processing (69.2% Milling Yield)	Extrusion: 98.2 °C preconditioner, 319.4 rpm shaft speed	Moderate	Decrease 15.3% fat, increase +6.6% starch.	Not assessed	Neutral				
	MFD (Mill-Feed) Bran-Rich Fraction Processing (28.5% Milling Yield)	Extrusion: 98.2 °C preconditioner, 319.4 rpm shaft speed	Intensive	Decrease 21.7%, decrease 24.7% starch.	Not assessed	Harmful				
Ferreira et al. 2024 [169]	Cold-Pressed Flaxseed Oil	Mechanical cold-pressing extraction at 100 °C then room temperature	Intensive	Eliminated (0%) protein, +162.4% fat, eliminated (0%) fiber, eliminated (0%) soluble fiber, +1.3% α-linolenic acid, +106.8% vitamin E, +206.2% γ-Tocopherol, −98.9% FRAP antioxidant activity, −99.7% DPPH antioxidant activity, −98.4% total phenolics, −99.3% flavonoids.	Not assessed	Harmful	Primary	Low	B	No control or minimal processing comparison.
	Defatted Flaxseed Flour	Cold-pressing by-product + grinding homogenization	Moderate	+74.7% protein, −75.5% fat, +5.6% fiber, +183.8% soluble fiber, −3.3% α-linolenic acid, −77.4% total vitamin E, −90.3% γ-Tocopherol, +68.2% FRAP antioxidant activity, +307.9% DPPH antioxidant activity, +43.8% total phenolics, +45.1% flavonoids.	Not assessed	Neutral				
Murray et al. 1998 [170]	Whole Egg Control (WE)	Drying	Minimal	22.3% protein, 13% fat, 44.5% starch, 6.2% total fiber, 3.8% calcium, 0.9% phosphorus, 10.2% ash.	Total tract digestibility protein 100%, fat 100%, organic matter 100%, dry matter 100%	Beneficial	Primary	Low	B	Lacked moderate methods for dose response.
	Rendered Beef + Extrusion (RMBM)	Rendering/extrusion	Intensive	−3.1% protein, +6.2% fat, −0.4% starch, +12.9% total fiber, no change in calcium/phosphorus, +1% ash.	Total tract digestibility protein 96.7%, fat 99.5%, Organic matter 97.8%, dry matter 96.7%	Harmful				
	Fresh/Raw Beef + Extrusion (FB)	Extrusion	Intensive	−11.7% protein, +10% fat, +2% starch, +3.2% total fiber, +5.3% calcium, −10.01% phosphorus, +4.9% ash.	Total tract digestibility protein 98.5%, fat 100.1%, organic matter 99.4%, dry matter 98%	Harmful				
	Rendered Poultry + Extrusion (PBPM)	Rendering/extrusion	Intensive	−6.3% protein, +3.8% fat, no change in starch, −4.8% total fiber, +2.6% calcium, −10.01% phosphorus, +2.9% ash.	Total tract digestibility protein 98.1%, fat 100.3%, organic matter 99.4%, dry matter 98.8%	Harmful				
	Defatted Soy Flour + Extrusion	Extrusion	Intensive	−4.5% protein, +8.5% fat, −3.8% starch, no change in total fiber, no change in calcium, −11.1% phosphorus, +2.9% ash.	Total tract digestibility protein 96.8%, fat 99.5%, organic matter 97.2%, dry matter 96.5%	Harmful				
	Fresh Poultry + Extrusion (FP)	Extrusion	Intensive	+0.09% protein, +19.2% fat, −3.6% starch, +12.9% total fiber, +2.6% calcium, −11.1% phosphorus, −1% ash.	Total tract digestibility protein 98.5%, fat 100.1%, organic matter 98.7%, dry matter 97.6%	Harmful				
Gong et al. 2015 [171]	Dehulled Barley	Boiling at 100 °C for 10 min	Moderate	Total phenolics 100%, total flavonoids 100%, free Ferulic Acid (0.379 mg/100 g DW), free amino acids (1.022 g/100 g DW), DPPH antioxidant (0.38 mg/g DW).	Baseline 100% digestibility	Neutral	Secondary	Low	B	No control or minimal processing comparison.
	Dehulled Barley	Steam explosion at 180 °C for 60 s, rapid pressure release	Intensive	Total phenolics +6.4%, total flavonoids +30.4%, free Ferulic Acid +119.8% (0.833 vs. 0.379 mg/100 g DW), free amino acids +158% (2.634 vs. 1.022 g/100 g DW), DPPH antioxidant +83.8% (0.69 vs. 0.38 mg/g DW), FRAP activity +32.5%.	+158% protein digestibility, −19.5% soluble carbohydrate digestibility, +119.8% free ferulic digestibility, +193.2% conjugated ferulic digestibility, +81.6% DPHH digestibility	Beneficial				
	Dehulled Barley	Extrusion at 180 °C, 400 rpm screw speed, 20 kg/h feed rate	Intensive	Total phenolics −0.04%, total flavonoids +12.5%, free Ferulic Acid +79.9% (0.833 vs. 0.379 mg/100 g DW), free amino acids +43.4% (2.634 vs. 1.022 g/100 g DW), DPPH antioxidant +34.2% (0.69 vs. 0.38 mg/g DW), FRAP +82.1%.	+43.4% protein digestibility, +0.05% soluble carbohydrate digestibility, +79.9% free ferulic digestibility, +29% conjugated ferulic digestibility, +134.2% DPHH digestibility	Beneficial				
	Dehulled Barley	Sand roasting at 240 ± 5 °C for 2 min in iron pan with 500 g sand	Intensive	Total phenolics −5.8%, total flavonoids −10.03%, free Ferulic Acid −15% (0.833 vs. 0.379 mg/100 g DW), free amino acids +8.8% (2.634 vs. 1.022 g/100 g DW), DPPH antioxidant +7.9% (0.69 vs. 0.38 mg/g DW), FRAP +11.6%.	+8.8% protein digestibility, −23.5% soluble carbohydrate digestibility, −15% free ferulic digestibility, −6.6% conjugated ferulic digestibility, +7.9% DPHH digestibility	Neutral				
Augustin 2024 [172]	Chickpea	Review article	Tempering, Roasting (wet or dry), dehulling, germination, milling, cleaning	Tempering + heating were more effective for decreasing phenolics/tannins. Tempering + heating increased in vitro-digestibility by 6%. Malting + heating increased protein digestibility.	Not assessed	Not assessed	Not assessed	Low	F	N/A
Machado 2023 [173]	Vegetable Oils	Review article	Heating	Exposure to heat, light, and metal in processing are known to increase reactive oxygen species and unpleasant taste/odors.	Not assessed	Not assessed	Not assessed	Low	F	N/A
Stone et al. 2021 [174]	Chickpea, Green Lentils, Navy Beans, Yellow Peas	Milled into flour	Minimal	Average protein 22.175%, average ash 3.225%, average lipid 35.325%.	Average in vitro protein digestibility 75.4%, average rapidly digestible starch content 8.8%	Neutral	Primary	Low	B	Lacked moderate methods for dose response.
	Chickpea, Green Lentils, Navy Beans, Yellow Peas	Roasting at 160 °C for 30 min, milled into flour, and 20% added moisture	Intensive	Average protein 22.4%, average ash 3.275%, average lipid 35.6%.	Average in vitro protein digestibility 79.5%, average rapidly digestible starch content 9.95%	Beneficial				
	Chickpea, Green Lentils, Navy Beans, Yellow Peas	Roasting at 160 °C for 30 min, milled into flour, and 30% added moisture	Intensive	Average protein 23.65%, average ash 3.25%, average lipid 35.55%.	Average in vitro protein digestibility 78.2%, average rapidly digestible starch content 13.38%	Beneficial				
Liu et al. 2020 [175]	High-amylose corn starch	Mixed with 7.82% water and cooked at 95 °C for 6 min	Moderate	Not assessed	Average rapidly digestible starch content 52.9%	Neutral	Secondary	Low	C	No control or minimal processing comparison. Narrow scope.
		Mixed with 7.82% water and cooked at 120 °C for 6 min	Moderate	Not assessed	Average rapidly digestible starch content 56.7%	Beneficial				
		Mixed with 7.82% water and cooked at 140 °C for 6 min	Intensive	Not assessed	Average rapidly digestible starch content 54.2%	Beneficial				
Napolitano et al. 2018 [176]	Hazelnuts	Skinned and crushed	Minimal	Fresh hazelnuts had a more diverse and complex lipid profile, rich in bioactive lipids.	Not assessed	Beneficial	Secondary	Low	C	Lacked moderate methods for dose response and narrow scope.
		Roasted at 170 °C for 30 min	Intensive	Roasted hazelnuts showed a decrease in some beneficial lipids (oxylipins, LCBs) but gained oxidized phospholipids, which could affect taste and shelf life.	Not assessed	Harmful				
DeVries 2020 [177]	Non-Starch Polysaccharides	Review article	Heat, mechanical energy, milling	The effects of enzyme addition on digestibility of the fiber fraction are 1.5–6 times larger, when applied to heat processed diets compared with unprocessed diets.	Not assessed	Not assessed	Not assessed	Low	F	No data to extract
Pilcoquesado et al. 2020 [178]	Quinoa	Milled into flour	Minimal	Protein 9.6%, lipid 15.2%, fiber 6.2%, ash 5.5%.	Not assessed	Neutral	Primary	Low	B	Lacked moderate methods for dose response.
		Soaked, germinated for 24 h, milled, roasted at 90 °C for 5 min	Intensive	Protein 15.6%, lipid 10.4%, fiber 6.2%, ash 2.6%.	Not assessed	Neutral				
		Soaked, germinated for 48 h, milled, roasted at 90 °C for 5 min	Intensive	Protein 18.6%, lipid 7.4%, fiber 6.2%, ash 3%.	Not assessed	Neutral				
		Soaked, germinated for 78 h, milled, roasted at 90 °C for 5 min	Intensive	Protein 26%, lipid 7.6%, fiber 7.4%, ash 4.5%.	Not assessed	Neutral				
	Kiwicha Seeds	Milled into flour	Minimal	Protein 15.4%, lipid 13.7%, fiber 7.5%, ash 3.8%.	Not assessed	Beneficial				
		Soaked, germinated for 24 h, milled, roasted at 90 °C for 5 min	Intensive	Protein 17.4%, lipid 9.3%, fiber 7.3%, ash 3.4%.	Not assessed	Neutral				
		Soaked, germinated for 48 h, milled, roasted at 90 °C for 5 min	Intensive	Protein 20.2%, lipid 7.9%, fiber 7.2%, ash 3.1%.	Not assessed	Neutral				
		Soaked, germinated for 78 h, milled, roasted at 90 °C for 5 min	Intensive	Protein 23.7%, lipid 5.4%, fiber 7.5%, ash 3.7%.	Not assessed	Neutral				
Lund et al. 2022 [179]	Whey Protein Isolates/Concentrates, Infant Formula	Pasteurization, spray drying, reverse osmosis, filtration, mixing	Not assessed	Processing increased Maillard reaction products and advanced glycation end products, which can lead to reduced digestibility and loss of lysine/arginine.	Not assessed	Not assessed	Not assessed	Low	F	No data to extract
Dhital et al. 2014 [180]	Basmati, Sushi, Jasmine, Low GI, and Vita Par Rice	Raw	Minimal	Not assessed	Average digestion extent 29.86% after 20 min, 54.88% after 120 min, and 69.3% after 240 min	Neutral	Primary	Low	B	Lacked intensive methods for dose response.
	Basmati, Sushi, Jasmine, Low GI, and Vita Par Rice	Boiling at 100 °C for 10 min with 30 rpm shaking	Moderate	Not assessed	Average digestion extent 72.8% after 20 min, 82.16% after 120 min, and 86.3% after 240 min	Beneficial				
Zhang et al. 2021 [181]	Rice Bran, Buckwheat, Wheat, Green Leaves, Corn, Pigmented Rice, Adlay, Black Rice, Ginger, Grape, Capsicum, Liver	Review article	No processing used in study design	Functional ingredients of wholegrains, including phenolic acids, cyanidin-3- glucoside, dietary fibers, zeaxanthin, lutein, phytanic acid, rutin, and octacosanol, positively affect the activity of brown adipocytes and browning of white adipocytes.	Not assessed	Not assessed	Not assessed	Low	F	No data to extract
Rizzi 2003 [182]	Corn, Processed Corn Products	Review article	Heating, pressure, pH	Heat treatment significantly affects DNA integrity. However, high amounts of amplifiable DNA are still present in the processed product.	Not assessed	Not assessed	Not assessed	Low	F	No data to extract
Triani and Foegeding 2019 [183]	Whey Protein Concentrates	Heating, water addition, pH adjustments	Not assessed	Time, temperature, and pH play vital roles in solubility, denaturation, and Maillard browning.	Not assessed	Not assessed	Not assessed	High	F	Unclear variables, lack of processing intervention, and narrow scope.

**Table 5:** Bayesian meta-analytic estimates: additives.

			95% CI		95% PI	
	Mean	Median	Lower	Upper	Lower	Upper
Pooled effect	5.965 × 10^−5^	0.000	0.000	0.001	−3.339 × 10^−4^	0.002
*τ*	1.202 × 10^−4^	0.000	0.000	0.001		
*τ* ^2^	8.153 × 10^−7^	0.000	0.000	1.504 × 10^−6^		
*I* ^2^	0.068	0.000	0.000	0.138		
H^2^	1.001	1.000	1.000	1.001		

**Table 6:** Bayesian effect size meta-regression terms test: additives.

Moderator	Dogs BF	Cats BF
Species	0.103	0.368
Additive type	0.119	0.396
Duration	0.066	0.49
Quality grade	0.117	0.441
RoB	0.158	0.552

**Table 7:** Meta-analytic estimates: Bayesian meta-analysis preservatives.

			95% CI		95% PI	
	Mean	Median	Lower	Upper	Lower	Upper
Pooled effect	2.996 × 10^−4^	0.000	−0.004	0.008	−0.006	0.009
*τ*	4.674 × 10^−4^	0.000	0.000	0.007		
*τ* ^2^	4.961 × 10^−6^	0.000	0.000	5.211 × 10^−5^		
*I* ^2^	0.23	0.000	0.000	2.646		
H^2^	1.003	1.000	1.000	1.027		

**Table 8:** Bayesian effect size meta-regression terms test: preservatives.

Moderator	Dogs BF	Cats BF
Species	0.229	0.434
Additive type	0.339	0.600
Duration	0.309	0.520
Quality grade	0.297	0.578
RoB	0.356	0.601

**Table 9:** Meta-regression estimates: impact of ingredient type on digestion.

			95% CI		95% PI	
	Subgroup	Estimate	Lower	Upper	Lower	Upper
Pooled effect	Chickpea	0.463	−40.462	41.388	−68.635	69.561
	Green lentil	3.584	−26.194	33.362	−44.436	51.603
	Navy bean	14.095	−69.595	97.785	−118.751	146.941
	Yellow pea	9.074	−11.909	30.057	−11.909	30.057
	Resistant starch (functional RS2 HA)	6.100	−0.155	12.356	−16.178	28.379
	Resistant starch (functional RS3 retro)	4.956	−1.788	11.701	−14.104	24.017
	Resistant starch (functional RS4 cross-linked)	3.751	0.964	6.539	−5.945	13.447
	Low GI brown rice					
	Basmati rice					
	Sushi rice					
	Jasmine rice					
	Vita parboiled rice					
	Whole rice	−1.325	−4.712	2.063	−8.506	5.856
	Beef byproducts	−0.722	−1.256	−0.188	−1.600	0.156
	Poultry byproducts	−0.376	−1.013	0.262	−1.855	1.104

**Table 10:** Weighted-regression test for funnel plot asymmetry: impact of processing on digestion.

	Asymmetry Test			Limit Estimate μ		
Estimates	*t*	df	*p*	Estimates	Lower 95% CI	Upper 95% CI
102	5.681	100	<0.001	−2.933	−3.392	−1.394

**Table 11:** Meta-analytic estimates: impact of ingredient type on nutrients.

			95% CI		95% PI	
	Subgroup	Estimate	Lower	Upper	Lower	Upper
Pooled effect	Pumpkin	0.511	−35.466	36.489	−60.021	61.044
	Butternut squash	−3.561	−10.642	3.520	−10.642	3.520
	Sweet potato	−4.582	−5.320	−3.845	−5.320	−3.845
	Drumstick leaves	1.003	−2.872	4.878	−2.872	4.878
	Pumpkin leaves	0.164	−81.070	81.397	−138.179	138.506
	Amaranth leaves	−7.091	−112.393	98.210	−184.734	170.551
	Finger millet	2.211	−5.031	9.454	−5.031	9.454
	Amaranth grain	−0.308	−27.040	26.425	−44.754	44.138
	Pigeonpea	−1.845	−33.702	30.013	−54.823	51.134
	Field bean	0.128	−23.184	23.440	−38.307	38.563
	Groundnut	−0.0820	−25.796	24.155	−42.084	40.443
	Pumpkin seed	−1.294	−28.826	26.237	−46.908	44.319
	Sunflower seed	1.284	−34.731	37.299	−59.159	61.727
	Whole soybeans in dog food 0%	0.620	0.254	0.986	0.254	0.986
	Whole soybeans in dog food 100%	7.110	−32.926	47.146	−55.238	69.458
	Whole soybeans in dog food 200%	30.728	−35.817	96.372	−35.817	96.372
	Whole soybeans in dog food 300%	48.691	−154.607	251.990	−248.869	346.251
	Black bean	−0.789	−1.165	−0.414	−2.036	0.457
	Chickpea	−0.027	−6.044	5.989	−11.019	10.965
	Green lentil	1.268	−3.095	5.631	−7.897	10.433
	Navy bean	0.821	−2.323	3.965	−6.809	8.452
	Yellow pea	3.510	−1.362	8.381	−8.489	15.508
	Quinoa	5.925	3.080	8.771	−1.236	13.087
	Kiwicha	5.635	3.193	8.078	−0.171	11.442
	Tibetan hull-less barley	2.974	1.166	4.782	−3.113	9.062

**Table 12:** Weighted regression test: impact of processing on nutrients.

	Asymmetry Test			Limit Estimate μ		
Estimates	*t*	df	*p*	Estimate	Lower 95% CI	Upper 95% CI
137	7.976	135	<0.001	−2.329	−3.039	−1.618

**Table 13:** Digestibility comparisons between human- and feed-grade diets.

	Hendriks et al. 2013 [188]	Oba et al. 2020 [152]
Parameter	Feed-Grade Diets (Ileal Cannulated Canines)	Human-Grade Diets (Cecectomized Rooster Assay)
Avg Dry Matter Digestibility	75.1% *	75.01% *
Avg Organic Matter Digestibility	79.4% *	81.8% *
Arginine	91.7% *	88.3–90.1%
Histidine	78.7% *	84.2–87.7%
Isoleucine	80.60%	85.1–88.3%
Leucine	84.4% *	86.3–89.4%
Lysine	81.60%	86.3–90.1% *
Methionine	83.80%	87.3–92.7% *
Phenylalanine	84.60%	85.2–88.1%
Threonine	79% *	79–83.6%
Valine	79.6% *	83–86.3%

Note. Numerical values followed by an “*” indicate that statistical significance was reached (*p* < 0.05).

## Data Availability

The data presented in this study are available on request from the corresponding author.
